# Crystal structure of the Bloom's syndrome helicase indicates a role for the HRDC domain in conformational changes

**DOI:** 10.1093/nar/gkv373

**Published:** 2015-04-21

**Authors:** Joseph A. Newman, Pavel Savitsky, Charles K. Allerston, Anna H. Bizard, Özgün Özer, Kata Sarlós, Ying Liu, Els Pardon, Jan Steyaert, Ian D. Hickson, Opher Gileadi

**Affiliations:** 1 Structural Genomics Consortium, University of Oxford, ORCRB, Roosevelt Drive, Oxford OX3 7DQ, UK; 2 Center for Chromosome Stability and Center for Healthy Aging, Department of Cellular and Molecular Medicine, University of Copenhagen, Panum Institute, Building 18.1, Blegdamsvej 3B, 2200 Copenhagen N, Denmark; 3 Structural Biology Brussels, Vrije Universiteit Brussel, Pleinlaan 2 , 1050 Brussels, Belgium; 4 Structural Biology Research Center, VIB, Brussel, Pleinlaan 2, 1050 Brussels, Belgium

## Abstract

Bloom's syndrome helicase (BLM) is a member of the RecQ family of DNA helicases, which play key roles in the maintenance of genome integrity in all organism groups. We describe crystal structures of the BLM helicase domain in complex with DNA and with an antibody fragment, as well as SAXS and domain association studies in solution. We show an unexpected nucleotide-dependent interaction of the core helicase domain with the conserved, poorly characterized HRDC domain. The BLM–DNA complex shows an unusual base-flipping mechanism with unique positioning of the DNA duplex relative to the helicase core domains. Comparison with other crystal structures of RecQ helicases permits the definition of structural transitions underlying ATP-driven helicase action, and the identification of a nucleotide-regulated tunnel that may play a role in interactions with complex DNA substrates.

## INTRODUCTION

Bloom's syndrome (BS) is an extremely rare autosomal recessive disorder that is characterized by shortness of stature, a distinctive skin rash and the predisposition to the development of a wide spectrum of cancers at an early age (1). Individuals with BS carry mutations in *BLM* that result either in expression of truncated proteins or alterations of key conserved residues within the RecQ helicase domain (1–3). On a cellular level, BS is characterized by excessive chromosome instability, including chromatid gaps and breaks (4), various chromosome structural rearrangements (5) and an increase in the number of sister chromatid exchange (SCE) events, the latter serving as a distinguishing feature for the clinical diagnosis of BS (6).

Bloom's syndrome helicase (BLM), like all RecQ-family helicases, acts as a 3′ to 5′ helicase (7) on a wide variety of DNA substrates including forked duplexes, G quadruplexes, 4-way junctions (8) and displacement loops (D loops) (9). It forms a multi protein complex with human topoisomerase IIIα (10), RMI1 (11) and RMI2 (12), termed the ‘dissolvasome’, which can promote the convergent branch migration (13) and decatenation of double Holliday junction intermediates formed during homologous recombination. This dissolution reaction prevents the exchange of genetic material flanking two homologous sequences engaged in homologous recombination (14,15).

**Figure 1.**
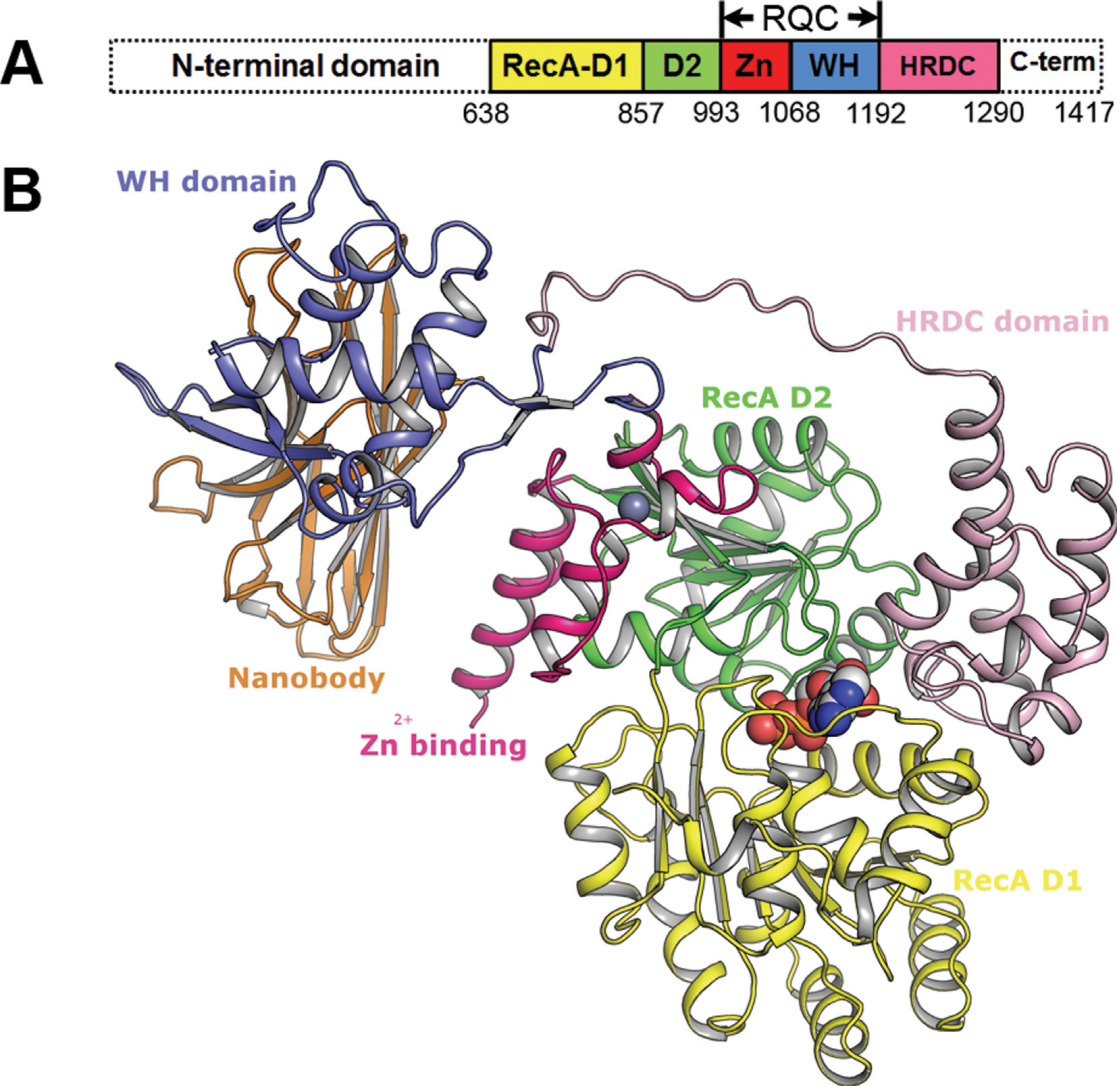
Structure of the BLM Nanobody complex. (**A**) Conserved domains in BLM protein. (**B**) Structure of the BLM nanobody complex with domains labelled and coloured as in (A), and the ADP and bound zinc ion are shown in the sphere representation. This figure and all subsequent molecular graphics figures were created using the program PyMOL (Schrödinger LCC).

The BLM protein is a 1417 amino acid, 159-kDa polypeptide with multiple structural domains (Figure 1A). At its centre (residues 639–1290) is a catalytic core conserved amongst RecQ-family helicases. A large N-terminal domain (residues 1–638) is believed to play a role in regulation and oligomerization of BLM (16,17), and is important in mediating interactions with partner proteins (10,18–19). The C-terminal region (residues 1291–1417), also involved in protein interactions (10), is believed to be predominantly unstructured, and contains a nuclear localization signal (20). We have focussed this study on the core domain, which is responsible for the basic functions of DNA unwinding.

The core domain itself is composed of subdomains, with varying degrees of evolutionary conservation (Figure 1A). The adenosine triphosphate (ATP)-dependent motor activity resides in two RecA-like domains termed D1 and D2 (residues 639–857 and 858–993, respectively), which contain sequence motifs shared amongst superfamily 2 (SF2) helicases (reviewed in (21)). Further downstream is the RecQ family-specific C-terminal domain (RQC) that includes a helical hairpin and Zn^2+^ binding subdomain (residues 994–1068, termed Zn) and a Winged Helix (WH) DNA binding domain (residues 1069–1192). Two of the β-strands in the WH domain form a prominent hairpin that has been suggested to act as a DNA strand separation pin in other RecQ helicases (22–24). Finally, residues 1193–1290 constitute the HRDC (helicase and RNase D C-terminal) domain, which occurs in two of the five human RecQ-family helicases (BLM and WRN), as well as in RecQ helicases in bacteria and yeast (25); as its name suggests, it is also present in other nucleic acid modifying enzymes including RNase D and RNA polymerase II subunit 4 (25,26). BLM mutants lacking the HRDC domain, whilst possessing core helicase and adenosine triphosphatase (ATPase) activities similar to wild-type protein (27–29), are defective in both strand annealing (27) and double Holliday junction dissolution (28). Moreover, a K1270V point mutation in the HRDC domain was found to affect the higher order functions of BLM, giving rise to a protein unable to dissolve double Holliday junctions efficiently (28), suggesting that the HRDC domain plays a crucial role during dissolution.

The structures of various HRDC domains have been determined in isolation (30–32). Despite the core structure being conserved, both the primary sequence and surface properties of the HRDC domains vary markedly, with the BLM HRDC surface being predominantly electronegative whilst the HRDC domain from WRN is predominantly electropositive (33). On the basis of these differences it has been suggested that the HRDC domain may play a role in modulating interactions with other components in protein complexes (32). On the other hand, it has been suggested that the HRDC domain binds to DNA (25). Indeed, numerous studies have implicated various HRDC domains with a direct role in DNA binding (30,31), although only very weak signle-stranded DNA (ssDNA) binding (*K*_d_ ∼ 100 μM) could be detected in one study for the BLM HRDC domain (34) whilst another similar study was unable to detect any binding when testing at concentrations up to 10 μM (33). Hence, the mechanism underlying HRDC function in dissolution remains obscure.

Determining the ways in which the structural domains of BLM relate and interact with each other will be the key to understanding the role of BLM in the maintenance of genome stability and how it is able to catalyze complex reactions such as double Holliday junction dissolution. In this study we present the 2.8-Å resolution crystal structure of BLM^636–1298^, in complex with both adenosine diphosphate (ADP) and a single domain llama antibody fragment (nanobody) specifically raised to BLM as an aid to crystallization. We also present two structures of BLM in complex with DNA at 4.7–3.5-Å resolution containing 12-bp duplex DNA with single-stranded 3′-overhangs. Comparisons to other RecQ helicase structures reveals the highly dynamic nature of the protein. Finally, we show that the HRDC domain packs tightly against the cleft formed between the two RecA-like domains D1 and D2 and that this binding is sensitive to the nucleotide status of the enzyme.

## MATERIALS AND METHODS

### Generation and selection of nanobodies

Nanobody CA5075 resulted from a feasibility study for generating and selecting nanobodies against a large number of antigens to align the process of Nanobody generation with other high-throughput approaches in proteomics, structural genomics and diagnostics (to be published elsewhere). In summary, 94 purified antigens, including eight membrane proteins, were collected from the Structural Genomics Consortium. All proteins were expressed in *Escherichia coli* and purified at the SGC protein production pipeline (35). Two non-inbred llamas were injected six times at weekly intervals with a mixture of all 94 proteins (50 μg of each antigen weekly). After six rounds of immunization, two separate phage display libraries were constructed, one from each animal. After pooling both libraries, nanobodies were selected against individual antigens in two rounds of parallel panning in 96-well plates containing one immobilized antigen in each well. After two rounds of selection by panning, 22 clones were picked from each selection for further characterization. For BLM, all 22 clones encoded antigen-specific Nanobodies as tested in ELISA, grouping in eight different sequence families. A Nanobody family is defined as a group of nanobodies with a high similarity in their CDR3 sequence (identical length and >80% sequence identity). Nanobodies from the same family derive from the same B-cell lineage and bind to the same epitope on the target. Immunizations, library construction, selection by panning and Nanobody characterization were performed according to standard procedures.

### Protein expression and purification

Nanobody CA5075 was subcloned to be expressed in *E. coli* with a PelB leader and a C-terminal His_6_ tag. The full protein sequence is indicated, with the asterisk indicating the point of cleavage during export to the bacterial periplasm.

MKYLLPTAAAGLLLLAAQPAMA*QVQLQESGGGLVQAGGSLRLSCAASGIWFSINNMAWYRQTPGKQRERIAIITSAGTTNYVDSVKGRFTISRDDAKNTMYLQMNSLIPEDTAVYYCNLVADYDMGFQSFWGRGTQVTVSSHHHHHH.

The protein was expressed in 3 l of *E. coli* grown in Terrific Broth with 0.1-mg/ml ampicillin at 37°C to OD = 3; the temperature was then lowered to 28°C. After 1 h, 0.2-mM Isopropyl β-D-1-thiogalactopyranoside (IPTG) was added, and incubation continued overnight at 180 RPM. The cells were collected by centrifugation and suspended in 100-ml HES buffer (200-mM 4-(2-Hydroxyethyl)piperazine-1-ethanesulfonic acid (HEPES), pH 7.5, 0.5-M sucrose, 0.5-mM ethylenediaminetetraacetic acid (EDTA)) and frozen at −80°C in 50-ml aliquots. Fifty millilitre of cell suspension was thawed and kept on ice for 1 h with occasional mixing. The cells were diluted with 50 ml of water. After another 1-h incubation on ice, the cells were removed by centrifugation and the protein was recovered from the supernatant (periplasmic extract). The protein was then purified by binding to Ni-IDA Sepharose, which was washed and eluted with 0.3-M imidazole then further purified by gel filtration in Superdex S75 in 50-mM HEPES, pH 7.5, 0.5-M NaCl, 5% glycerol.

BLM ^636–1298^ (spanning the helicase, RQC and HRDC domains) was expressed in *E. coli* and purified by Ni-Sepharose, tag cleavage, heparin-Sepharose and Superdex 200 as described in the Supplementary information of (36). The heparin step was later abandoned as it did not provide further purification.

BLM^636–1072^, including the RecA domains, was cloned into vector pNIC-Zb (Genbank GU452710 (35)), which appends a His_6_ tag and a highly basic Zb sequence for increased yield and purity. The proteins were expressed in *E. coli* and purified by Ni-affinity, ion exchange on SP-Sepharose, digestion with Tobacco Etch Virus (TEV) protease and gel filtration on Superdex S75. The mutants H666A and S729A were generated by site-directed mutagenesis and purified using the same method.

BLM^1206–1298^, encoding the HRDC domain, was cloned into pCDF-BirA (Genbank JF914075 (37)), which appends a TEV-cleavable His_10_ tag at the N-terminus and a biotin acceptor sequence at the C-terminus. The protein was expressed in *E. coli* in TB medium supplemented with 20-mg/l biotin and purified using Ni-IDA affinity capture and gel filtration on S75 Superdex column. The K1270V mutant was similarly purified.

#### Full-length BLM proteins

The BLM(S666A) and BLM(S729A) variants were generated from pJK1-BLM(WT) by a site-directed mutagenesis strategy. The resulting pJK1- BLM(S666A) and pJK1-BLM(S729A) were transformed in *Saccharomyces cerevisiae*-strain JEL1. Cell growth, protein induction and purification were performed as described in (7) with the exception that the nickel affinity chromatography was performed using a HisTrap FF 1-ml column (GE Healthcare) according to the manufacturer′s recommendations. Eluted proteins were dialyzed overnight against the storage buffer (50-mM Hepes pH7.5, 1-mM DTT, 0.1-mM EDTA, 200-M NaCl, 10% glycerol). The concentration of the different purified BLM variants was determined by Coomassie Blue staining using bovine serum albumin (BSA) as a standard.

### Crystallization and structure determination

BLM protein precipitated in low-salt buffers unless supplemented with glycerol or other stabilizing additives. For co-crystallization with nanobodies, the BLM and nanobodies were mixed in GF buffer (10-mM HEPES, pH 7.5, 500-mM NaCl, 5% glycerol, 0.5-mM TCEP) and the complexes were purified by gel filtration (Superdex 200) in the same buffer. ADP and MgSO_4_ were added to 0.5 mM each. The proteins were then concentrated to 17 mg/ml and crystallization was set up with standard crystallization screens supplemented with 15% glycerol. Crystals for the BLM Nanobody complex were obtained from conditions comprising 0.1-M MES pH 6.0, 22% PEG 20000 and glycerol and were cryoprotected by soaking in a solution consisting of the well solution supplemented with 15% glycerol. Crystals were loop mounted and plunged into a pool of liquid nitrogen. Diffraction data extending to 2.8-Å resolution were collected on beamline I24 at Diamond light source. The diffraction data were processed with the program XDS (38) and belong to a primitive trigonal crystal system, space group P1. The structure was solved by molecular replacement with a domain-based search strategy, using the program PHASER (39) and the structure of human RecQ1(22), for positioning of the D1, D2, Zinc binding and WH domains, the structure of the BLM HRDC domain (PDBid 2RRD) (33) and a nanobody fragment from the PDBid 3EZJ. Overall the electron density map is of high quality given the resolution throughout the two BLM molecules and two nanobodies in the asymmetric unit. All of the residues of BLM are present in the final model with the exception of the first three residues at the N-terminus and eight at the C-terminus, and two loops spanning residues 1004–1016 and 1093–1106 which are disordered. Thus the final model consists of 1490 residues out of a theoretical total of 1620, and has been refined to a crystallographic *R*_factor_ of 20.4% (*R*_free_ = 24.7%), with no residues in the outlying regions of the Ramachandran plot.

Preparation of oligonucleotide duplexes was performed as outlined above. DNA complexes were prepared by mixing BLM (15 mg/ml) and DNA in a molecular ratio of 1:1.1 with ADP/Mg^2+^ added at a final concentration of 1 mM in a buffer containing 4-mM HEPES, 200-mM NaCl, 16.7% glycerol. Crystals of the BLM DNA complex I were obtained from conditions comprising 21% PEG 4000, 0.1-M HEPES pH 7.6, 5% isopropanol. Crystals of BLM-DNA complex II were obtained from well solutions comprising 19% PEG 4000, 0.1-M Hepes pH 7.2, 5% isopropanol. Both crystals were cryoprotected by soaking in a solution consisting of the well solution supplemented with 23% ethylene glycol, before being loop mounted and plunged into a pool of liquid nitrogen. Data sets extending to ∼3.2-Å resolution (complex I) and 4.8-Å resolution (complex II) were collected on beamline I24 at Diamond light source. Data were processed with XDS (38), and the structure was solved by molecular replacement using the program PHASER (39) and the BLM nanobody complex as a search model, with the DNA being modelled manually into the electron density maps after several rounds of refinement using the program COOT (40). Using a conventional measure of crystallographic data quality (I/SigI for the highest resolution shell of 2.0) the nominal resolution of the complex I data set would be 3.5 Å, however the highly anisotropic nature of this data meant that significant strong data extending to beyond 3.2 Å were present for data along the crystallographic l-axis, inclusion of which resulted in significant improvements in electron density maps. Thus the final refinement was performed using data to 3.2 Å with the program phenix.refine (41), using the BLM nanobody complex used as a reference model for restrained refinement of complex I (*R*_factor_ 0.235, *R*_free_ of 0.273), and using a rigid body refinement strategy for complex II (*R*_factor_ of 0.235, *R*_free_ of 0.297). In both cases it was noted that a significant improvement of the electron density maps was obtained by B-factor sharpening, presumably a result of the large Wilson B factor and consequently high B-factors in the models which down weight the high-resolution terms in the map calculation. B-factor sharpening was implemented using a sharpening B-factor determined automatically by phenix refine. A full summary of the data processing and refinement statistics is shown in Supplementary Table S1.

### Helicase assays

The fork and 4-way junction substrates were prepared as described in (8). One femtomole of substrate was incubated in the presence of the indicated amount of BLM in a 10-μl reaction mixture containing 50-mM Tris-HCl pH7.5, 1-mM DTT, 5-mM ATP, 3-mM MgCl_2_, 90-mM NaCl and 200-μg/ml BSA. Reactions were incubated 15 min at 37°C and stopped by the addition of 0.2% sodium dodecyl sulphate, 10-mM EDTA and 100-μg/ml Proteinase K. After 30 min of incubation at 37°C, reaction products were separated with 12% acrylamide gel at 15 V/cm constant for 1 h at room temperature. Gels were dried and exposed overnight on a phosphor-imaging screen. The image was recorded using a Typhoon scanner.

### Double Holliday junction (dHJ) dissolution assays

dHJ substrate was prepared as described in (42). Reactions were carried out as described for the helicase assays but in the presence of 1 fmole of dHJ substrate and 5 nM of TopoIIIα/Rmi1/Rmi2 (TopoIIIα/Rmi1/Rmi2 purification will be described elsewhere). Prior to loading, reaction products were heated at 80°C for 2 min and immediately cooled on ice for 5 min. Reaction products were separated with 8% native acrylamide gel electrophoresis run at 4°C.

### ATPase assays

ATPase activity was measured in 50-mM Tris pH 7.5, 50-mM NaCl, 5-mM MgCl_2_, 1-mM DTT, at 37°C for 10 min. The reaction mixtures contained 0.5-mM ATP, 500-nM single-stranded DNA (50-mer oligonucleotide) and 1–100-nM BLM. The reactions were stopped, and the amount of hydrolyzed ATP was monitored by the malachite green method, using a commercial kit (Innova Biosciences).

### SCE analysis

To check the ability of two mutants to correct SCE frequency in BS cells; GM08505 cells, an SV40-transformed fibroblast cell line from a BS patient, were transfected with wt or mutant forms of BLM cDNA in pcDNA3 plasmid, as described previously (43). The cells were then selected with 600-μg/ml G418 (Sigma Aldrich). PSNG13 and PSNF5 cell lines with an empty vector and a Flag epitope-tagged BLM protein, respectively, (43) were used as controls. SCE analysis was performed as previously described (44), with the following modifications: phosphate buffered saline (pH 6.8) was used instead of Sorensen's phosphate buffer and the final staining was done in 5% Giemsa.

### Biolayer interferometry

Binding kinetics of RecA domains to HRDC domain were determined by the biolayer interferometry (BLI) method (45) using the Octet RED384 instrument (FortéBio). Biotinylated HRDC domain was immobilized on Streptavidin biosensors that were subsequently used for association and dissociation experiments. Each experiment was performed for a 60-s window in a buffer containing 20-mM HEPES pH7.5, 200-mM NaCl and 5% glycerol. The signals from HRDC-coated and biotin-blocked control biosensors were measured in the same experiment.

After reference correction, the subtracted signals from HRDC-coated biosensors were analysed in the FortéBio analysis software provided with the instrument.

### Small angle X-ray scattering

Small angle scattering measurements of BLM in solution were performed at Diamond light source beamline B21 using a BIOSAXS robot for sample loading. Measurements were made using three different protein concentrations: 13, 6.5 and 3.25 mg/ml, in the presence or absence of 1-mM ADP, ATP or ATPγS. The data were reduced and buffer subtraction was performed with the DawnDiamond software suite. Data quality was evaluated using the ATSAS software suite and P(r) functions were calculated using GNOM (46). Scattering profiles of atomic models were calculated using CRYSOL (47) and aligned and scaled to the experimental data using PRIMUS (48) and data fits were evaluated using the Chi-free (49) calculation in the SCATTER package (www.BIOSIS.NET). Rigid body modelling was performed using the program SASREF (50).

## RESULTS

### Crystal structures of BLM-nanobody and BLM-DNA complexes

Despite repeated attempts, we were unable to crystallize BLM in the absence of stabilizing agents. For this reason we used BLM-specific nanobodies as an aid to crystallization (51). In parallel, numerous DNA molecules were tested, and a 3′-tailed duplex DNA yielded diffracting co-crystals with BLM. ADP was required for successful crystallization in both cases.

Crystals of the BLM^636–1298^-ADP-nanobody complex diffracted to 2.8-Å resolution, and the structure was solved by molecular replacement. Examination of the crystallographic contacts provides a rationale for the role of the nanobody in promoting crystallization, with specific inter- and intra-molecular contacts mediating crystal packing (Supplementary Figure S1A). The overall architecture of BLM is a large globular core, formed primarily by the two RecA domains, to which the Zn^2+^ binding and HRDC domains are closely associated (Figure 1B). The WH domain projects away from this globular region, forming no contacts with the rest of the protein, interacting instead with the nanobody molecule. An examination of the crystal contacts does not provide any indication of higher order oligomerization, consistent with the finding that BLM constructs lacking the extended N-terminal region are monomeric (52).

Crystals of the same BLM fragment in complex with ADP and DNA partial duplexes diffracted to lower resolution (3.5 Å). The structures of the individual domains of BLM are virtually identical between the nanobody and DNA complexes (Supplementary Figure S1B). However, the relative orientation of the WH domains is markedly different, with a rotation of 90° required to place the domains in equivalent positions. The WH domain in the DNA complex structure is in a similar position to that observed for human RECQ1 (HsRECQ1 (22,53)) and, as described below, is poised to play a similar role in DNA strand separation. We assume, therefore, that the position of the WH domain in the nanobody complex is different due to the absence of the DNA and, possibly, its stabilization by the nanobody.

### The RecA domains and nucleotide binding

**Figure 2.**
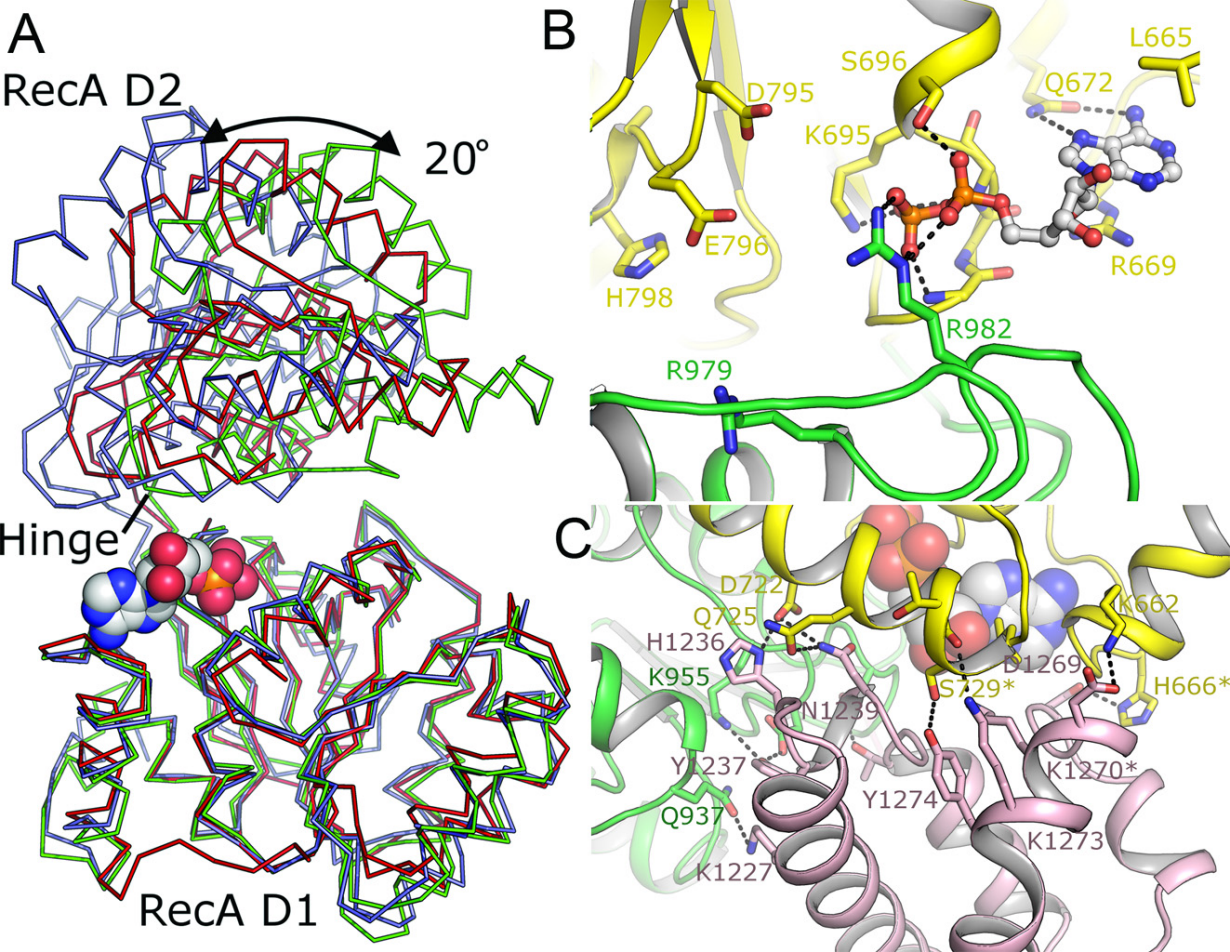
Structural details of the nucleotide binding site and the HRDC. (**A**) Comparison between BLM (green), human RECQ1 (blue) and *E. coli* RecQ (red) structures superposed on the basis of the D1 domain reveals conformational differences in the relative positioning of the D1-D2 domains, which affect the distance between D2 and the nucleotide bound to D1. (**B**) Close-up view of the nucleotide binding site with the ADP moiety in the ball and stick format and key interacting residues and motifs labelled. (**C**) Close-up view of the interface between the HRDC domain and the two RecA domains, with polar contacts highlighted. Residues highlighted with an asterisk were mutated to test their effect on BLM activity.

The individual fold of the two RecA domains is very similar to the HsRECQ1 and *E. coli* RecQ (EcRecQ) structures (22,54), with RMSD's between equivalent Cα atoms of 1.5 Å. However, the relative positioning of these two domains differs significantly in all three structures, with the BLM and HsRECQ1 being the most different and EcRecQ being somewhat intermediate (Figure 2A). The inter-domain movement can be described as a rigid body shift of up to 20°, with the loop connecting the two domains (residues 855–858) acting as a hinge, resulting in maximal displacements of equivalent regions of up to 15 Å. The cleft between the two RecA domains is considerably narrower in BLM than in either EcRecQ or HsRECQ1, with the D1-D2 conformation in BLM crystals appearing to be quite distinct amongst the SF2 family helicase structures solved to date.

The ADP moiety binds in the cleft between the two RecA domains and makes extensive interactions with the D1 domain (Figure 2B). Although Mg^2+^ was included in the crystallization cocktail, it is not seen in the structure. The ADP nucleotide is bound to the D1 domain in a very similar arrangement to that seen in HsRECQ1 and EcRecQ. The adenine base of the ADP lies between the side chains of L665 and R669 and makes hydrogen bonds to the main chain of N667 and a bivalent hydrogen bond to Q672 (Figure 2B). These residues are part of the ‘helicase motif 0’ (55) and are crucial for conferring nucleotide triphosphate specificity to RecQ helicases. The ribose O4 makes a single hydrogen bond to R669, whilst the α- and β-phosphates form multiple polar contacts to the glycine-rich loop that belongs to the widely conserved helicase motif I or ‘Walker A’ motif.

Only two residues in the D2 domain, which are part of helicase motif VI, are in close proximity to the ADP. R982 has been proposed to act as an ‘arginine finger’ contributing to ATP hydrolysis through stabilization of a transition state, and to the coupling of ATP hydrolysis to conformational changes in the protein (56,57). In our structure, two guanidine nitrogens of R982 are able to donate hydrogen bonds to both the α-and β-phosphates. Indeed, R979, another motif VI residue that is important for BLM ATPase/helicase activity, lies close to the expected position of the γ-phosphate and (assuming a different side-chain rotamer) would also be able to form polar contacts with it (Supplementary Figure S2). We therefore suggest that the coordinated activities of both R982 and R979, either of which may contact the γ-phosphate during different stages of the catalytic cycle, may play a role in energy coupling. Notably, these two residues are not in close contact with the ADP in the structure of HsRECQ1 (22), in which the D1 and D2 domains are most distant.

### Conformation of the Zn^2+^ binding and WH domains

The Zn^2+^ binding domain is well conserved in both its overall structural features and its relative positioning (Supplementary Figure S1C) across the three RecQ family structures, differing mainly in the length of the helical hairpin (BLM > HsRECQ1 > EcRecQ).

As noted previously (22,24,58), the main-chain structures of the WH domains of EcRecQ, HsRECQ1, WRN and BLM are very similar (RMSD's ∼2 Å for 97 aligned cα atoms), despite having very low sequence identity. The most prominent difference is in the β-hairpin region, which is shortest in EcRecQ, of intermediate length in WRN, and longest in HsRECQ1 and BLM (*ibid*).

### Structure of the BLM-DNA complex

In order to gain insight into the mode of BLM DNA binding we have determined the crystal structure of BLM, in the absence of a nanobody, but in complex with ADP and two different DNA oligonucleotides, containing 12 bp of duplex DNA with either a 5 or a 12 nucleotide 3′ overhang. The best diffraction (3.5-Å resolution) was obtained for BLM DNA crystals with the 5 nucleotide overhang (complex I); crystals containing a 12 nucleotide overhang (complex II) diffracted less well (4.5-Å resolution).

**Figure 3.**
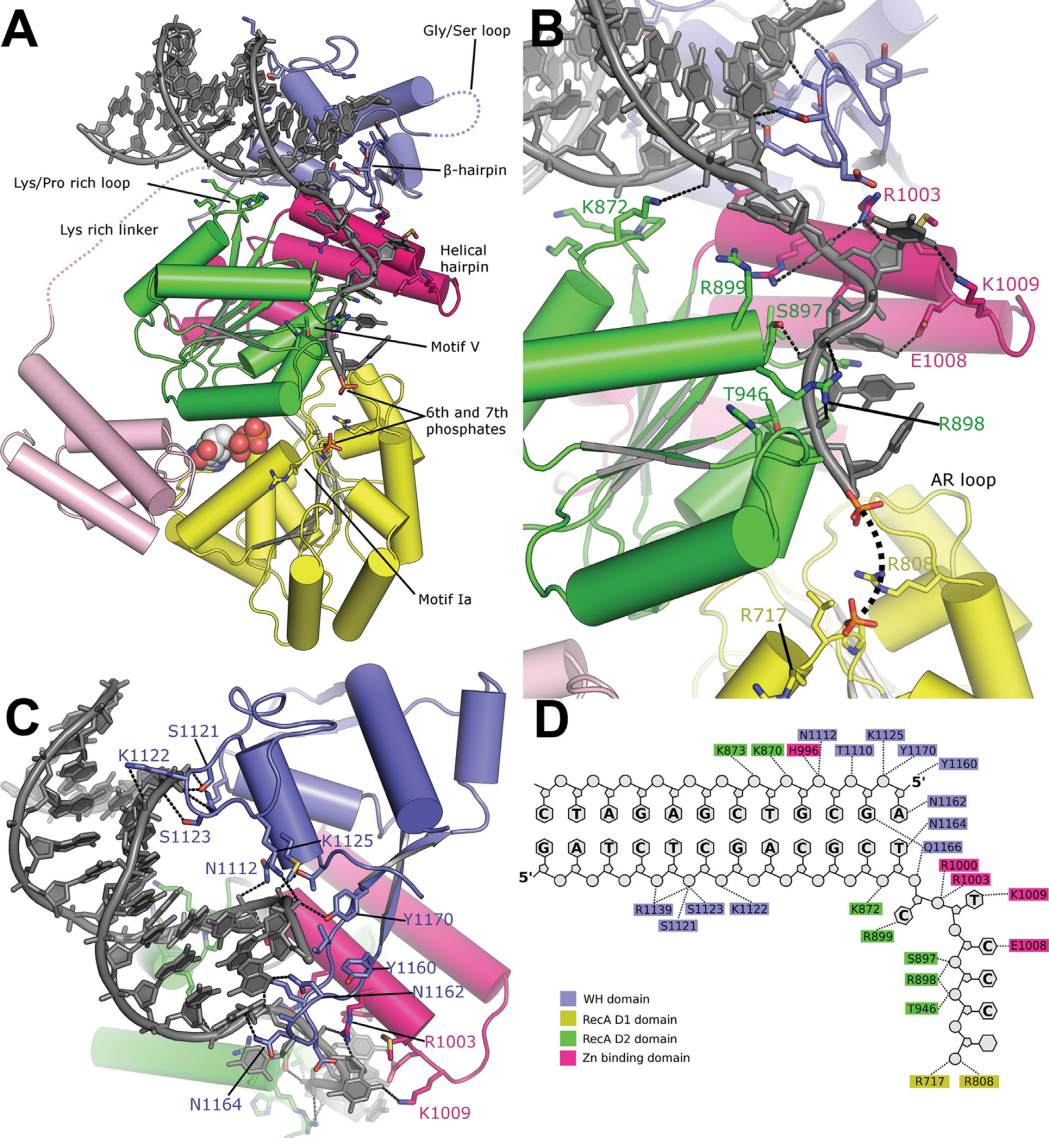
The structure of the BLM-DNA complex. (**A**) Overview of the BLM-DNA complex with key structural features involved in positioning of the DNA labelled and the expected path of missing loop regions indicated by dashed lines. (**B**) Close-up of the interface between the WH domain and the double-stranded DNA. (**C**) The interaction of the 3′-single-stranded overhang with BLM. The sixth and seventh phosphates are shown in stick representation in the positions revealed in the low-resolution structure of the BLM-DNA complex II. (**D**) A schematic summary of BLM-DNA contacts.

In both DNA complex crystals the blunt ends of the DNA form stacking interactions with a symmetry mate around a crystallographic 2-fold symmetry axis. Clear electron density can be observed in complex I for the entire length of the DNA (Supplementary Figure S3A), the duplex region of which is positioned between the D2 domain, the Zn^2+^ binding domain and the WH domain (Figure 3A). The majority of the contacts to the duplex DNA are formed via the WH domain, which contacts DNA primarily via the N-terminus of the second helix and the extended loop between the second and third helices, forming an approximately equal number of polar contacts to each strand (Figure 3B and D).

Consistent with a role in strand separation (22,23), the β-hairpin on the WH domain is positioned at the interface between the double- and single-stranded regions on the minor groove face (Figure 3A). Whilst the role of the β-hairpin appears to be well conserved amongst RecQ family members, its sequence has diverged significantly with both the presence and positioning of aromatic residues serving as distinguishing features. In both HsRECQ1 and WRN, aromatic residues within the β-hairpin form stacking interactions with bases at the interface (22,24,53). In contrast, the β-hairpin of BLM lacks these aromatic residues, instead forming an extensive and predominantly polar interface with the final paired base of the duplex region (Figure 3A and B).

A strand separating hairpin is a common feature of DNA helicases of superfamily 2 (HsRECQ1, BLM, WRN and archaeal HEL308) as well as SF1 (e.g. PcrA). However, the overall geometry of the interaction with DNA differs markedly (Supplementary Figure S3B). In BLM the DNA duplex is almost perpendicular to the 3′ overhang, whilst in the HsRECQ1 DNA complex it is almost parallel. HsRECQ1 resembles HEL308, whilst BLM is more similar to the WRN WH-DNA complex ((24); not shown). This may be of critical importance for the differential substrate specificity of the two proteins (59). The positioning of the dsDNA also allows an interaction with a region outside the WH domain: a lysine/proline-rich loop (residues ^868^PKKPKK^873^) that connects the first β-strand to the first α-helix of the D2 domain. Two residues at the tip of this loop K870 and K872 form salt bridges with the phosphodiester backbone. HsRECQ1 lacks these residues and instead contains a longer loop that would make steric clashes with the DNA if similarly positioned. A summary of protein–DNA contacts is depicted in Figure 3D.

The five bases of the 3′ overhang stretch from immediately after the β-hairpin and pass close to the helical hairpin of the Zn^2+^ binding domain to contact the D2 domain (Figure 3C). The first three bases on the 3′ overhang are flipped out, pointing in the opposite direction to their predecessors, in a manner that resembles the flipped bases in the SF1 helicase PcrA (60). Evidence in support of this base-flipping mechanism comes from the fact the both BLM and WRN helicases show significantly inhibited helicase activity on substrates with restricted DNA backbone flexibility (61), a trait that is shared by PcrA, but not the majority of other SF2 helicases.

The phosphate of the second base in the overhang is positioned in a basic pocket formed by a cluster of arginine side chains R899, R1000 and R1003, the latter two are part of the helical hairpin region of the Zn^2+^ binding domain, which can also be seen to transition from disordered to ordered upon DNA binding.

The trajectory of the ssDNA overhang continues in an arrangement that is highly conserved in other helicase–DNA complexes (60,62); the fourth and fifth phosphates make polar contacts with the conserved S897 and R898 of helicase motif IV and T946 of motif V (Figure 3C).

An indication of the further trajectory of the ssDNA is provided by examination of difference electron density maps of complex II, which reveals two significant positive peaks (between 5 and 6 σ), that are appropriately spaced (∼7.5 Å) to correspond to the positions of the sixth and seventh phosphates of the 3′ overhang (Supplementary Figure S3C). Due to the low resolution of this data set, only phosphate positions have been modelled for these two bases, and contacts to these phosphates are inferred from the proximity of the respective groups rather than directly observed in the electron density. The phosphate of the sixth base is in an intermediate position between the D2 and D1 domains and does not interact with the rest of the protein. In contrast, the seventh base of the 3′ overhang is closely associated with the D1 domain, close to the conserved helicase motif Ia. The side chains of R717 (part of motif Ia) and R808 (part of the conserved ‘aromatic-rich’ loop) are sufficiently close to provide favourable electrostatic interactions with the phosphate oxygens (Figure 3C and Supplementary Figure S3C). The conserved helicase motif Ia has previously been identified through mutational analysis as a single-stranded DNA binding motif in SF2 helicases (63), and mutation of the equivalent of R808 in the aromatic-rich loop of EcRecQ to leucine results in an enzyme that is unable to efficiently couple ATP hydrolysis to helicase activity (64). The additional five bases present in the DNA of complex II were not visible in the electron density, indicating that in the current ADP-bound conformation, D2 is the only domain that binds ssDNA tightly.

### Conformation of the HRDC domain and linker region

The HRDC domain has been found to be dispensable for the core helicase activity of BLM (29). The HRDC domain itself is very similar to the solution structures of BLM HRDC determined in isolation (1.0- and 1.4-Å RMSD, respectively, (33,34)). Somewhat surprisingly, the HRDC domain packs, via its electropositive face, against a shallow cleft between the two RecA domains close to the adenine and ribose groups of the bound ADP (Figure 2C). The interface is particularly polar in nature and contains 14 hydrogen bonds (10 to the D1 domain and 4 to D2) and three salt bridges (1 to D1 domain and two to D2). Several of the residues forming the interface are well conserved in sequence alignments of BLM homologues (Supplementary Figure S1D). Of particular interest is residue K1270, which was previously shown to be essential for efficient double Holliday junction dissolution (28), being involved in both hydrophobic and polar interactions with the D1 domain. The HRDC domain is linked to the WH domain by an extended lysine-rich linker (residues 1199–1208), which extends away from the rest of protein, creating a large hole in the surface (Figure 1B and Supplementary Figure S6).

### Solution studies of HRDC–D2 interactions

To examine the validity of the HRDC-core interaction and its implications on the activity of BLM, we performed binding experiments with the isolated domains, as well as small-angle X-ray scattering (SAXS), and functional analysis *in vitro* and in cells. Based on the reported functional deficiencies of the K1270V mutant, which lies in the HRDC–D1 interface, we also generated two mutants in the D1 domain (H666A and S729A) that are also part of this interface, and tested their functional consequences.

**Figure 4.**
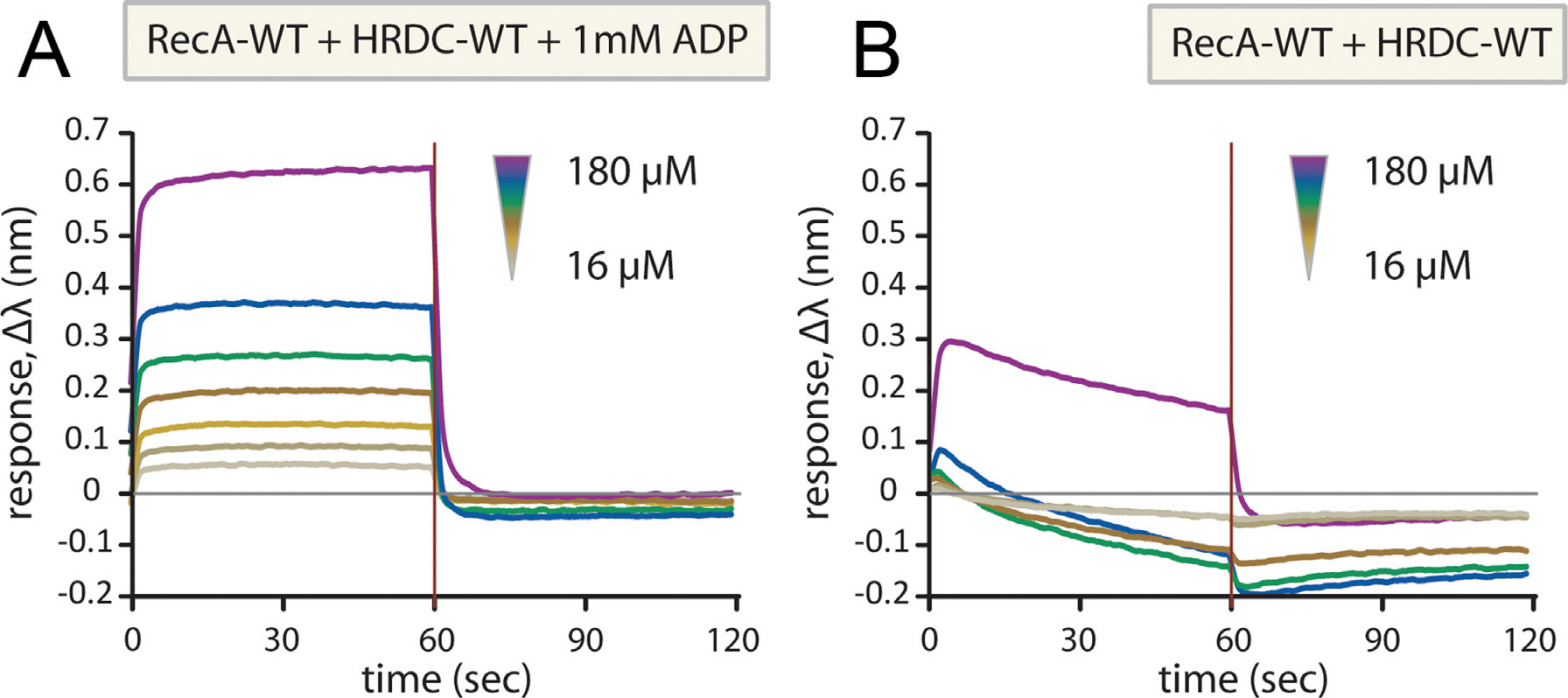
Investigation of the interaction of isolated HRDC domain (residues 1206–1298) with the two RecA domains (residues 636–1072) by BLI. The biotinylated HRDC domain was immobilized on the tips, which were then dipped in solutions of the RecA-domains in the concentration range indicated, in the presence (**A**) or absence (**B**) of ADP.

To measure directly the interaction between the HRDC and RecA domains in solution, we measured the kinetics of this association using individually expressed domains by Biolayer interferometry. Despite problems associated with significant non-specific binding to the sensor tip, we were able to confirm an interaction between the HRDC domain and the RecA core in the presence of either ADP or ATP with an apparent affinity of 30-100 μM (Figure 4A and Supplementary Figure S4). Despite the moderate affinity, this interaction is likely to be significant in the native protein, where a flexible linker tethers the two domains. Crucially, this interaction could not be observed in the absence of nucleotide (Figure 4B), suggesting that D1 and D2 only form a competent interface for the binding of the HRDC domain in the presence of nucleotide. The interaction was severely affected by mutations in the predicted interaction surfaces on either the HRDC (K1270V) or the D1 (H666A and S729A) domain (Supplementary Figure S4), indicating that the binding occurs through the interface observed in the crystal structure.

**Figure 5.**
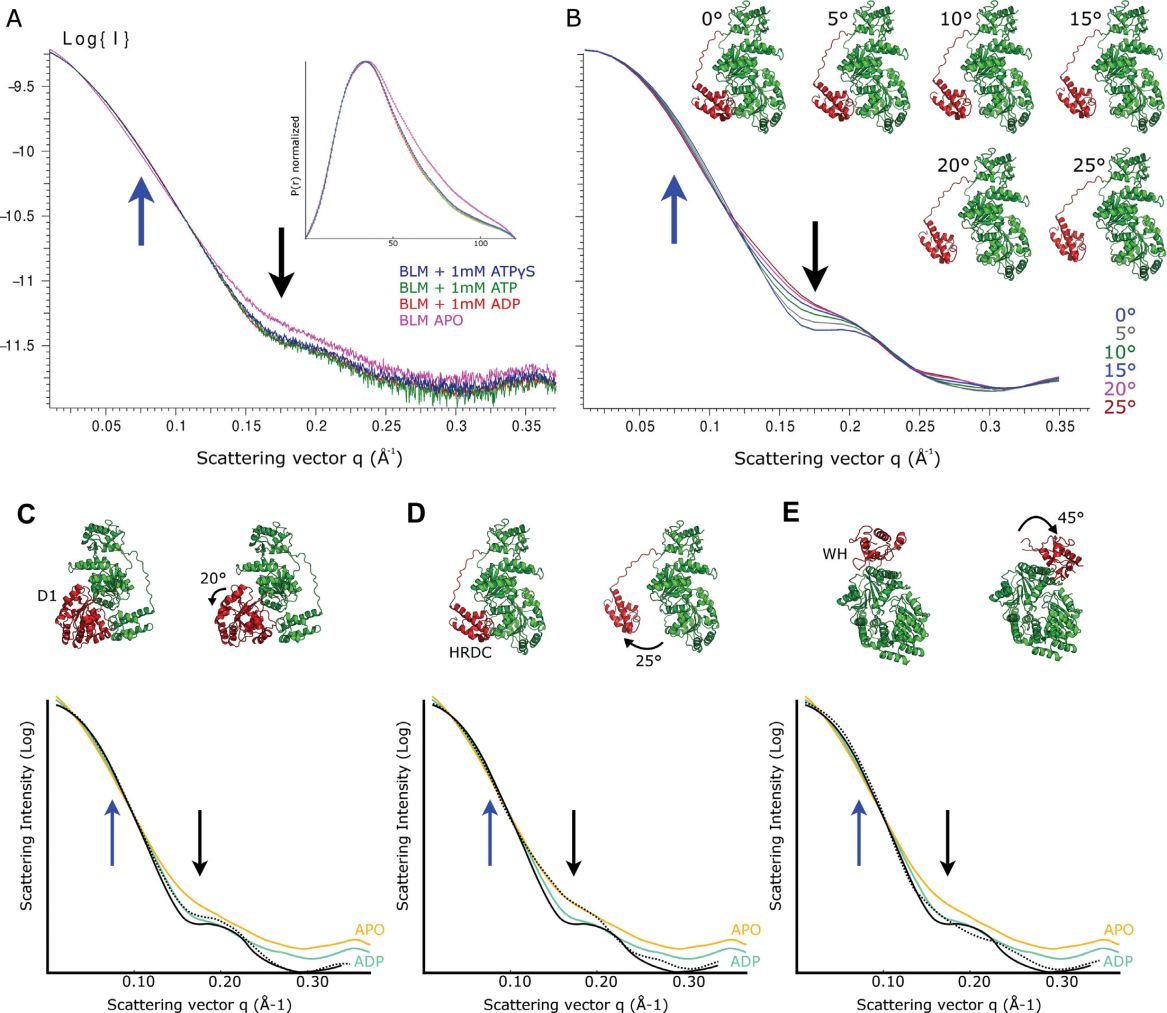
SAXS measurements and interpretation. (**A**) Experimental SAXS curves for various BLM nucleotide complexes with the corresponding pair distance distribution p(r) shown in the inset. Blue and black arrows highlight the scattering at *q* = 0.075 and 0.175 Å^2^, respectively. (**B**) Calculated scattering curves for various models with the HRDC and linker region (shown in red) rotated by the indicated angle. (**C–E**) Comparison of experimental and theoretical SAXS curves for three hypothetical types of domain movements. The solid black line represents the calculated curve for BLM in the BLM DNA complex (contribution of the DNA is omitted) and the dashed line represents the calculated curve following the domain movement. For the experimental data only the APO- and ADP-bound forms are shown and are fitted with a smoothed curve for clarity. (C) Movements of the relative positioning of the D1-D2 domain by up to 20° (based on the conformation of human RECQ1). (D) Movements of the HRDC domain and the linker region by 25°. (E) Movements of the WH domain (based on the conformation of the WH domain in *E. coli* RecQ).

To gain insight into conformational changes that may occur during the catalytic cycle, we measured the SAXS profiles of BLM^636–1298^ in the presence and absence of the nucleotides ADP, ATP and the ATP analogue ATPγS (Figure 5A). Whilst the scattering curves of the three nucleotide complexes are very similar, they all show significant differences when compared to the scattering of the apo protein, with a characteristic reduction of scattering intensity at *q* values of 0.075 Å^−1^ and an increase in scattering intensity at *q* values around 0.175 Å^−1^ (Figure 5A, marked with blue and black arrows, respectively). These differences are also reflected in a quantitative evaluation of SAXS curve similarity with the program Vr (65), which shows the apo protein is significantly more different than any two of the nucleotide-bound complexes (Supplementary Figure S5A).

To interpret these changes we have adopted a modelling approach, in which the experimental data were compared to calculated data from models in which the HRDC domain has been rotated to various degrees around a hinge where the linker meets the WH domain. Whilst such models are probably over-simplified, the general features and their magnitude (a reduction of scattering at *q* ≈ 0.075 Å^−1^ and an increase around *q* ≈ 0.175 Å^−1^ when the HRDC moves away from the RecA domains), as well as the overall shape, are in good agreement (Figure 5B). Alternative possibilities for conformational changes include the relative positioning of the D1 and D2 domains or even the WH domain. We note that a slightly better fit to the data is obtained for models where the WH domain is in the conformation adopted in the nanobody structure (Chi^2^/Chi_free_^2^ of 3.95/5.12, compared to 11.9/16.2 for the conformation of the DNA-bound structure) (Supplementary Figure S5B), suggesting the conformation BLM in the Nanobody crystals is a reasonable approximation of the nucleotide-bound DNA free structure. The flexibility of the various domains may also play a significant role in the scattering in solution, and analysis of the various experimental curves by the Porod–Debye law (66) reveals although generally compact, a slight increase in flexibility can be seen in the nucleotide free protein (Porod exponent decreases from ∼3.9 to 3.6). Clearly the situation in solution may be more complex than our model analysis with the potential for flexibility or multiple simultaneous domain movements to occur. Significantly when comparing the calculated scattering profiles for these various movements, only the movement of the HRDC domain accounts for the characteristic differences observed for the experimental data upon nucleotide binding (Figure 5C–E). This conclusion is generally supported by rigid body modelling using the program SASREF (REF) which when given the ability to move the HRDC domain becomes significantly distant from the core when refined against the APO data but not the nucleotide-bound data (Supplementary Figure S5C).

### Enzymatic activities of BLM mutants

**Figure 6.**
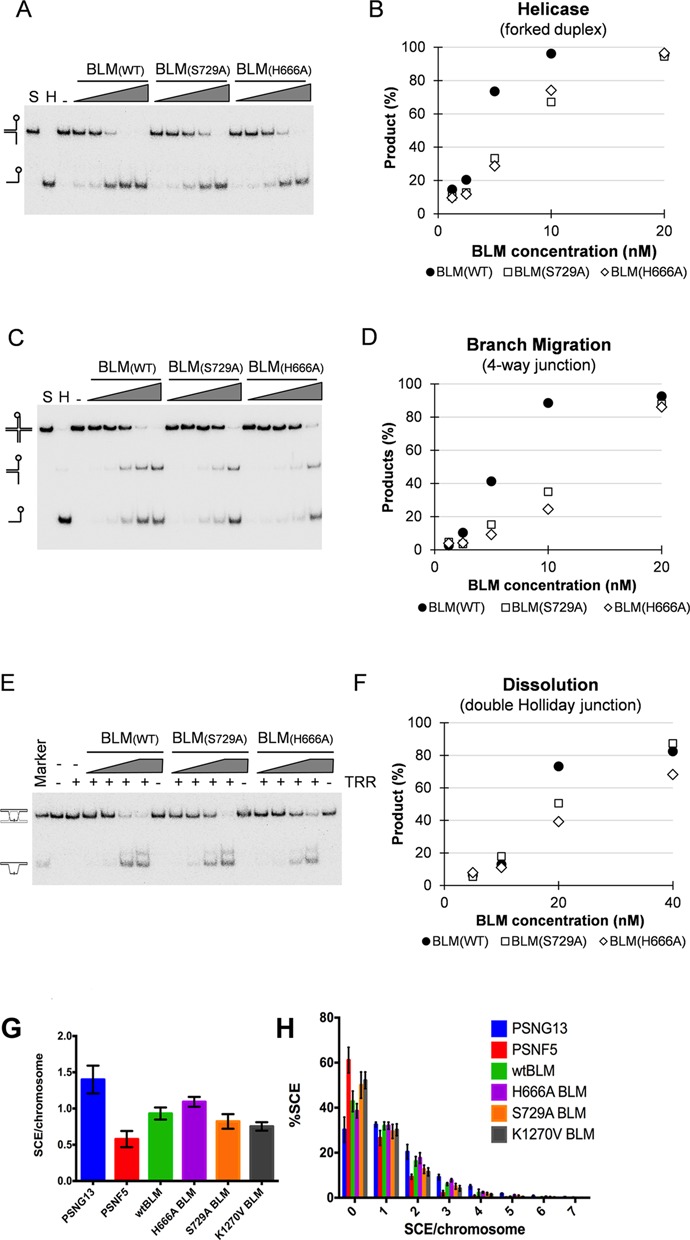
Activities of the HRDC/RecA-core interaction defective mutants BLM(S729A) and BLM(H666A). For each assay, a representative gel and its associated quantification are shown. (**A**) Helicase efficiency assayed on a forked duplex substrate in the absence (−) or in the presence of increasing concentration of BLM(WT), BLM(S729A) or BLM(H666A). (**B**) Quantification of helicase assay shown in (A). (**C**) Branch migration efficiency assayed on a 4-way junction substrate in the absence (−) or in the presence of increasing concentration of BLM(WT), BLM(S729A) or BLM(H666A). (**D**) Quantification of branch migration assay shown in (C). (**E**) dHJ dissolution efficiency assayed on a short synthetic dHJ junction substrate in the absence (−) or in the presence of increasing concentration of BLM(WT), BLM(S729A) or BLM(H666A) and in the absence (−) or in the presence (+) of TopoIIIα/Rmi1/Rmi2 (TRR). (**F**) Quantification of dissolution assay shown in (E). (**G-H**) All three mutantsH666A, S729A and K1270V were able to suppress the enhanced sister chromatid exchange frequency in BS cells, indicating that the mutant proteins are functional in a cellular context. GMO8505 cells, an SV40 immortalized BS cell line, were transfected with wt or mutant BLM. PSNG13 (BLM-) and PSNF5 (BLM+) were used as controls. The number of SCE per chromosome (**G**) and the distribution of SCE frequency per chromosome (**H**) were plotted for each sample. Data represent at least 3 experiments, with error bars showing the standard deviation. For each experiment, a minimum of 500 chromosomes per sample were counted.

Given the significantly lower dissolution activity of the K1270V mutant (28) and that the K1270A, H666A and S729A mutant proteins are all defective in the HRDC–RecA interaction, we tested the influence of H666A and S729A mutations on the enzymatic activities of full-length BLM. Both H666A and S729A mutants are functional for all biochemical activities tested, including DNA-stimulated ATPase, helicase, 4-way junction branch migration and double Holliday junction dissolution (Figure 6A–F). The mutants display a slight reduction in DNA-stimulated ATPase activity (4 s^−1^, 3 s^−1^ and 2.5 s^−1^, for WT, H666A and S729A, respectively) as well as in helicase and dissolution efficiency. The H666A and S729A mutants appear to be more defective (up to 3-fold) when assayed for 4-way junction branch migration. Finally, the ability of all three mutants to correct the enhanced SCE frequency of BS cells *in vivo* was very similar to that of WT BLM (Figure 6G-H), indicating that the mutant proteins were functional in a physiologically context.

### Comparisons with other BLM structures

**Figure 7.**
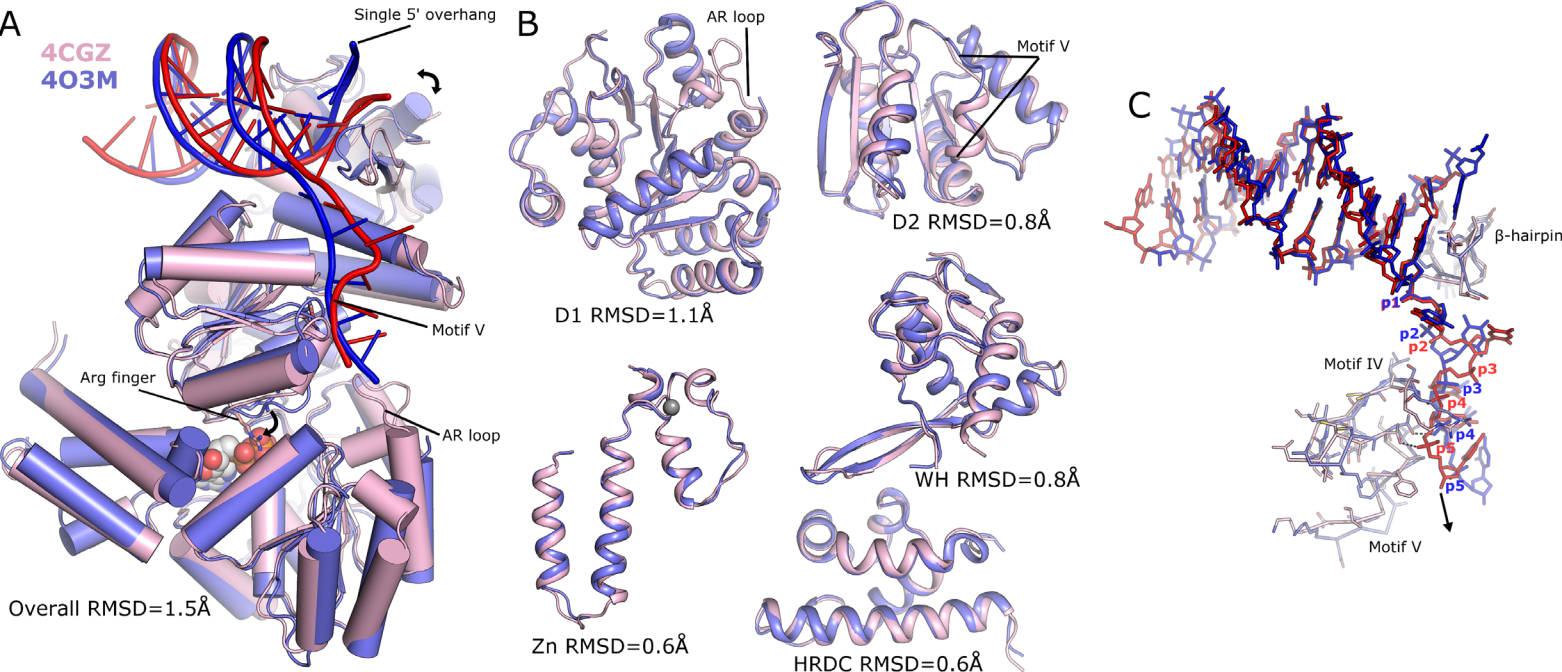
Comparison of the BLM-DNA complex (4CGZ) with 4O3M. (**A**) Overall comparison of the BLM-DNA complex (pink with red DNA) and 4O3M (blue with dark blue DNA), with the same view as in Figure 3A. (**B**) Comparison of individual domains with key structural differences labelled. (**C**) Comparison of the DNA substrates aligned by superposition of the WH domain. The conserved motifs IV and V on 4CGZ contact phosphates p4 and p5, whereas in 4O3M they make similar contacts to p3 and p4, consistent with a conformation that represents pre- and post-translocation states, respectively.

During the late stages of preparation of this manuscript another study was published in which the authors describe a structure of BLM in complex with ADP and DNA (PDB: 4O3M; (67)). This structure, determined at 2.3-Å resolution obtained in the presence of a 16-bp DNA duplex with an 8-bp overhang, is very similar to our DNA complex, and can be aligned with a 1.5-Å RMSD over the entire structure (Figure 7A). Comparing the individual domains separately reveals an even higher degree of similarity, indicating a slight difference is present in their relative positioning (Figure 7B). Superposition on the basis of the D1 domain alone reveals a small difference in WH domain orientation (maximal displacements of ∼4.5 Å between equivalent residues), which is possibly a result of the different lengths of double-stranded DNA present.

Despite these slight differences the β-hairpin forms a very similar conformation in both structures with a single additional unpaired Guanine base on the 5′ end being visible in 4O3M, which makes polar contacts to N1164 in the β-hairpin. The flipped orientation of the first base of the 3′ overhang is well conserved as are many of the polar contacts to the DNA (Figure 7A). The most prominent and significant difference lies in the number of nucleotides between β-hairpin and the conserved phosphate contacts on the D2 domain, with 4O3M containing three bases and our DNA complex containing four (Figure 7C). As a consequence of this, the DNA overhang in 4O3M is significantly straighter than in our DNA complex, and more distant from the helical hairpin motif, which is also partially disordered in 4O3M. It is tempting to speculate that these structures may correspond with a different conformational state of the crystallized enzyme, with 4CGZ and 4O3M being consistent with pre- and post-translocation states, respectively. Further support for this suggestion comes from differences observed in the conformation and ordering of residues in the vicinity of the nucleotide binding site. Both R979 and R982 of the ‘arginine finger motif’ are pointing away from the nucleotide in 4O3M, possibly due to the fact that a Ca^2+^ is bound to the expected position of the Mg^2+^ ion. In addition, residues 798–808, which form the aromatic-rich loop, are disordered in that structure (4O3M) and the helical region of helicase motif V adopts a significantly different conformation. These differences are particularly significant given that these structural motifs have been found to play a role in energy coupling (56–57,64).

## DISCUSSION

The five RecQ-family helicases in humans (RECQ1, BLM, WRN, RECQ4 and RECQ5) have catalytic cores with high sequence and structural similarity, yet their physiological roles *in vitro* substrate preferences, and catalytic properties vary considerably. Structural studies are required to fully understand both the common mechanisms and the basis for selectivity, and may contribute to developing small-molecule inhibitors (36,68). In this study, we combined structural information from co-crystal structures of the BLM catalytic core with a nanobody and with DNA to provide insights on its mechanisms of action. Many features of the interaction of BLM with ADP and with the 3′ ssDNA overhang are similar to those observed in other SF2 helicases, involving residues of the conserved helicase motifs. Within this framework, the relative positioning of the two RecA domains is crucial for understanding the catalytic cycle; the two domains are closer in our structure than in other published structures of RecQ helicases. In addition, the arginine side chains of motif VI (from domain D2) are positioned near the β and possibly the γ phosphate of the ATP. The structure may represent an intermediate pre- or post-hydrolysis stage within the catalytic cycle. It is interesting to note that the structures of both BLM and of HsRECQ1, which differ by an ∼20° relative rotation of the two RecA domains, contain bound ADP. This may relate to the kinetic observation (69) of a rate-limiting transition between two ADP-bound states in the catalytic cycle of BLM.

The interaction of the WH domain with duplex DNA and the positioning of the β-hairpin at the ds-ssDNA junction are in line with observations of the WH domains of WRN and human HsRECQ1 (53). Interestingly, although lacking an aromatic residue at the tip of the β-hairpin that is essential in HsRECQ1, the β-hairpin in BLM seems to have a similar structural role in stabilizing or forcing strand separation. However, the exact trajectory of the DNA varies markedly between BLM, WRN and HsRECQ1, and could form a critical determinant of the preferences for different DNA structures. The very divergent location of the WH domains between the different structures of BLM is in line with recent crystal and solution structures of Cronobacter sakazakii RecQ (70), as well as the earlier crystal structures of *E. coli* RecQ (54), indicating that the mobility of the WH domain is a general feature of RecQ helicases.

The most remarkable feature to emerge from this study is the new insight on the role of the HRDC domain. Whilst this domain has been variously hypothesized to interact with DNA or with other proteins, the crystal structure indicates a robust intramolecular association of the HRDC domain with the catalytic core, in a position to directly impact on the ATPase cycle. The distance of the HRDC domain from the DNA in our structure indicates that a direct DNA binding role is unlikely, although it remains a possibility for DNA substrates with higher complexity that those used in this study. The isolated HRDC and ATPase domains bind directly in solution with modest affinity (30-100 μM); this binding is dependent on the presence of nucleotide (ADP or ATP), and is disrupted by mutations in residues lying in the interface in the crystal structure.

Further evidence for a role of the HRDC domain in conformational changes comes from our SAXS analysis of BLM^636–1298^, which shows significant conformational changes associated with nucleotide binding and release. These changes are consistent with theoretical calculations of the scattering curves from models in which the HRDC domain is associating and dissociating from its binding cleft between the D1 and D2 domains. These findings are consistent with two roles of the intramolecular interaction (which are not mutually exclusive). First, the HRDC domain may influence the mechanochemical coupling of the ATPase cycle, perhaps playing a role in ensuring the correct relative positioning the single-stranded DNA binding regions of the D1 and D2 domains. Alternatively, the cycle of association and dissociation of the HRDC domain with nucleotide binding and release may control a more global aspect of the catalytic cycle. An intriguing possibility relates to the extended loop connecting the HRDC and WH domains which protrudes from the rest of the BLM surface, creating a tunnel-like structure when the HRDC is bound. The proximity of the blunt end of the DNA duplex to this loop leads us to suggest that the threading of ssDNA through this loop and its subsequent association with the HRDC domain may be a feature of BLM activity on complex substrates (in the context of the dissolvasome) such as during the late stages of the dissolution process (Supplementary Figure S6).

We have attempted to investigate the role of the HRDC–D1/D2 interaction in the biochemical and cellular activities of BLM by generating structure-guided mutations affecting the binding interface. We were prompted by a previous study, identifying the K1270V HRDC mutation, which affected double Holliday junction dissolution activity (28). Two residues in the D1 domain, H666 and S729, form hydrogen bonds with the HRDC domain; these residues are conserved in BLM orthologues in many species (Supplementary Figure S7) but less so in other RecQ-like and SF2 helicases (see (53)). Mutations of these residues (H666A, S729A) significantly reduced the binding of the HRDC domain in BLI experiments, but seemed to have only a marginal effect on enzymatic activity or on complementation of chromosomal instability in BLM-deficient cells. This is not entirely surprising given the previous finding of significant activity in BLM mutants lacking the entire WH and HRDC domains (29), and perhaps indicates that these domains only confer a significant evolutionary advantage within the context of a particular cellular pathway. We note that the binding affinity of the wild-type HRDC and D1/D2 domains measured by BLI is close to the detection limit in these experiments, such that a 2–3-fold decrease in affinity may lead to un-detectable binding. Thus, the extent of the defect in the two mutations may be modest, and they may still exhibit biochemically significant binding when tethered. Whilst it is possible that the HRDC–D1/D2 interaction is of no biological consequence, we suggest that more drastic alterations in structure are likely to be needed to change catalytic function.

**Figure 8.**
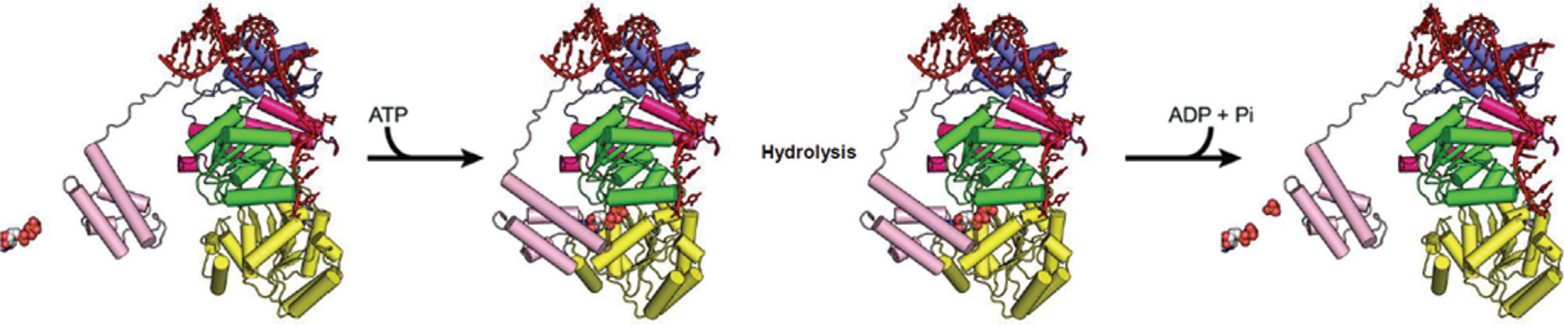
Model of the coupling of ATP hydrolysis/DNA translocation cycle with HRDC-mediated conformational change. We assume that in the absence of nucleotide the HRDC domain is disengaged, and the two RecA domains adopt a different relative orientation in which the distance between ssDNA binding motifs on D1 and D2 is approximately equal to the phosphate to phosphate distance of one nucleotide step (which in our model is based on the conformation of HsRECQ1). Nucleotide binding and hydrolysis is accompanied by the association of the HRDC domain, the adoption of the conformation of D1 and D2 found in the BLM crystal structures and the transition of the DNA from the pre-translocation state (found in our structure), to the post-translocation state based on PDB: 4O3M.

Further evidence for a role of the HRDC domain in the catalytic function of BLM comes from a recent structural and biochemical study in which an enzymatic characterization of BLM mutants lacking the entire HRDC domain was performed (67). In general agreement with our results it was found that BLM mutants lacking the HRDC domain had a lower *V*_max_ for DNA unwinding, higher *K*_m_ for DNA and a generally less efficient coupling of ATP hydrolysis to DNA unwi6nding (67). This study also included a structure of BLM in complex with DNA that is very similar to our DNA complex, although a detailed comparison indicates that it may correspond to a slightly different state of the crystallized enzyme. Based on these data we have attempted to construct a model for the BLM helicase mechanism and associated conformational changes that occur during the BLM catalytic cycle (Figure 8 and Supplementary Movie S1). However, it is clear that structural information on the nature of the interactions between BLM, topoisomerase IIIα, RMII and RMI2 (the ‘dissolvasome’) is required to understand the role of BLM in double Holliday junction dissolution and the maintenance of genome integrity. The structure and mechanism of BLM, presented above, can provide a framework from which these investigations can proceed.

## ACCESSION NUMBERS

PDB IDs: 4CGZ and 4CDG.

## SUPPLEMENTARY DATA

Supplementary Data are available at NAR Online.

SUPPLEMENTARY DATA

## References

[1] German J., Sanz M.M., Ciocci S., Ye T.Z., Ellis N.A. (2007). Syndrome-causing mutations of the BLM gene in persons in the Bloom's Syndrome Registry. Hum. Mutat..

[2] Guo R.B., Rigolet P., Ren H., Zhang B., Zhang X.D., Dou S.X., Wang P.Y., Amor-Gueret M., Xi X.G. (2007). Structural and functional analyses of disease-causing missense mutations in Bloom syndrome protein. Nucleic Acids Res..

[3] Kaneko H., Orii K.O., Matsui E., Shimozawa N., Fukao T., Matsumoto T., Shimamoto A., Furuichi Y., Hayakawa S., Kasahara K. (1997). BLM (the causative gene of Bloom syndrome) protein translocation into the nucleus by a nuclear localization signal. Biochem. Biophys. Res. Commun..

[4] German J., Archibal R., Bloom D. (1965). Chromosomal breakage in a rare and probably genetically determined syndrome of man. Science.

[5] Chu W.K., Hickson I.D. (2009). RecQ helicases: multifunctional genome caretakers. Nat. Rev. Cancer.

[6] Chaganti R.S., Schonberg S., German J. (1974). A manyfold increase in sister chromatid exchanges in Bloom's syndrome lymphocytes. Proc. Natl. Acad. Sci. U.S.A..

[7] Karow J.K., Chakraverty R.K., Hickson I.D. (1997). The Bloom's syndrome gene product is a 3′-5′ DNA helicase. J. Biol. Chem..

[8] Mohaghegh P., Karow J.K., Brosh R.M. Jr, Bohr V.A., Hickson I.D. (2001). The Bloom's and Werner's syndrome proteins are DNA structure-specific helicases. Nucleic Acids Res..

[9] Bachrati C.Z., Borts R.H., Hickson I.D. (2006). Mobile D-loops are a preferred substrate for the Bloom's syndrome helicase. Nucleic Acids Res..

[10] Wu L., Davies S.L., North P.S., Goulaouic H., Riou J.F., Turley H., Gatter K.C., Hickson I.D. (2000). The Bloom's syndrome gene product interacts with topoisomerase III. J. Biol. Chem..

[11] Wu L., Bachrati C.Z., Ou J., Xu C., Yin J., Chang M., Wang W., Li L., Brown G.W., Hickson I.D. (2006). BLAP75/RMI1 promotes the BLM-dependent dissolution of homologous recombination intermediates. Proc. Natl. Acad. Sci. U.S.A..

[12] Singh T.R., Ali A.M., Busygina V., Raynard S., Fan Q., Du C.H., Andreassen P.R., Sung P., Meetei A.R. (2008). BLAP18/RMI2, a novel OB-fold-containing protein, is an essential component of the Bloom helicase-double Holliday junction dissolvasome. Genes Dev..

[13] Karow J.K., Constantinou A., Li J.L., West S.C., Hickson I.D. (2000). The Bloom's syndrome gene product promotes branch migration of holliday junctions. Proc. Natl. Acad. Sci. U.S.A..

[14] Bizard A.H., Hickson I.D. (2014). The dissolution of double Holliday junctions. Cold Spring Harb. Perspect. Biol..

[15] Wu L., Hickson I.D. (2003). The Bloom's syndrome helicase suppresses crossing over during homologous recombination. Nature.

[16] Janscak P., Garcia P.L., Hamburger F., Makuta Y., Shiraishi K., Imai Y., Ikeda H., Bickle T.A. (2003). Characterization and mutational analysis of the RecQ core of the bloom syndrome protein. J. Mol. Biol..

[17] Xu Y.N., Bazeille N., Ding X.Y., Lu X.M., Wang P.Y., Bugnard E., Grondin V., Dou S.X., Xi X.G. (2012). Multimeric BLM is dissociated upon ATP hydrolysis and functions as monomers in resolving DNA structures. Nucleic Acids Res..

[18] Bergeron K.L., Murphy E.L., Brown L.W., Almeida K.H. (2011). Critical interaction domains between bloom syndrome protein and RAD51. Protein J..

[19] Srivastava V., Modi P., Tripathi V., Mudgal R., De S., Sengupta S. (2009). BLM helicase stimulates the ATPase and chromatin-remodeling activities of RAD54. J. Cell Sci..

[20] Hayakawa S., Kaneko H., Fukao T., Kasahara K., Matsumoto T., Furuichi Y., Kondo N. (2000). Characterization of the nuclear localization signal in the DNA helicase responsible for Bloom syndrome. Int. J. Mol. Med..

[21] Vindigni A., Marino F., Gileadi O. (2010). Probing the structural basis of RecQ helicase function. Biophys. Chem..

[22] Pike A.C., Shrestha B., Popuri V., Burgess-Brown N., Muzzolini L., Costantini S., Vindigni A., Gileadi O. (2009). Structure of the human RECQ1 helicase reveals a putative strand-separation pin. Proc. Natl. Acad. Sci. U.S.A..

[23] Lucic B., Zhang Y., King O., Mendoza-Maldonado R., Berti M., Niesen F.H., Burgess-Brown N.A., Pike A.C., Cooper C.D., Gileadi O. (2011). A prominent beta-hairpin structure in the winged-helix domain of RECQ1 is required for DNA unwinding and oligomer formation. Nucleic Acids Res..

[24] Kitano K., Kim S.Y., Hakoshima T. (2010). Structural basis for DNA strand separation by the unconventional winged-helix domain of RecQ helicase WRN. Structure.

[25] Morozov V., Mushegian A.R., Koonin E.V., Bork P. (1997). A putative nucleic acid-binding domain in Bloom's and Werner's syndrome helicases. Trends Biochem. Sci..

[26] Meka H., Daoust G., Arnvig K.B., Werner F., Brick P., Onesti S. (2003). Structural and functional homology between the RNAP(I) subunits A14/A43 and the archaeal RNAP subunits E/F. Nucleic Acids Res..

[27] Cheok C.F., Wu L., Garcia P.L., Janscak P., Hickson I.D. (2005). The Bloom's syndrome helicase promotes the annealing of complementary single-stranded DNA. Nucleic Acids Res..

[28] Wu L., Chan K.L., Ralf C., Bernstein D.A., Garcia P.L., Bohr V.A., Vindigni A., Janscak P., Keck J.L., Hickson I.D. (2005). The HRDC domain of BLM is required for the dissolution of double Holliday junctions. EMBO J..

[29] Gyimesi M., Harami G.M., Sarlos K., Hazai E., Bikadi Z., Kovacs M. (2012). Complex activities of the human Bloom's syndrome helicase are encoded in a core region comprising the RecA and Zn-binding domains. Nucleic Acids Res..

[30] Bernstein D.A., Keck J.L. (2005). Conferring substrate specificity to DNA helicases: role of the RecQ HRDC domain. Structure.

[31] Killoran M.P., Keck J.L. (2008). Structure and function of the regulatory C-terminal HRDC domain from Deinococcus radiodurans RecQ. Nucleic Acids Res..

[32] Liu Z., Macias M.J., Bottomley M.J., Stier G., Linge J.P., Nilges M., Bork P., Sattler M. (1999). The three-dimensional structure of the HRDC domain and implications for the Werner and Bloom syndrome proteins. Structure.

[33] Sato A., Mishima M., Nagai A., Kim S.Y., Ito Y., Hakoshima T., Jee J.G., Kitano K. (2010). Solution structure of the HRDC domain of human Bloom syndrome protein BLM. J. Biochem..

[34] Kim Y.M., Choi B.S. (2010). Structure and function of the regulatory HRDC domain from human Bloom syndrome protein. Nucleic Acids Res..

[35] Savitsky P., Bray J., Cooper C.D., Marsden B.D., Mahajan P., Burgess-Brown N.A., Gileadi O. (2010). High-throughput production of human proteins for crystallization: the SGC experience. J. Struct. Biol..

[36] Nguyen G.H., Dexheimer T.S., Rosenthal A.S., Chu W.K., Singh D.K., Mosedale G., Bachrati C.Z., Schultz L., Sakurai M., Savitsky P. (2013). A small molecule inhibitor of the BLM helicase modulates chromosome stability in human cells. Chem. Biol..

[37] Keates T., Cooper C.D., Savitsky P., Allerston C.K., Phillips C., Hammarstrom M., Daga N., Berridge G., Mahajan P., Burgess-Brown N.A. (2012). Expressing the human proteome for affinity proteomics: optimising expression of soluble protein domains and in vivo biotinylation. New Biotechnol..

[38] Kabsch W. (2010). Xds. Acta Crystallogr. D.

[39] McCoy A.J., Grosse-Kunstleve R.W., Adams P.D., Winn M.D., Storoni L.C., Read R.J. (2007). Phaser crystallographic software. J. Appl. Crystallogr..

[40] Emsley P., Lohkamp B., Scott W.G., Cowtan K. (2010). Features and development of Coot. Acta Crystallogr. D.

[41] Afonine P.V., Grosse-Kunstleve R.W., Echols N., Headd J.J., Moriarty N.W., Mustyakimov M., Terwilliger T.C., Urzhumtsev A., Zwart P.H., Adams P.D. (2012). Towards automated crystallographic structure refinement with phenix.refine. Acta Crystallogr. D.

[42] Bachrati C.Z., Hickson I.D. (2009). Dissolution of double Holliday junctions by the concerted action of BLM and topoisomerase IIIalpha. Methods Mol. Biol..

[43] Gaymes T.J., North P.S., Brady N., Hickson I.D., Mufti G.J., Rassool F.V. (2002). Increased error-prone non homologous DNA end-joining–a proposed mechanism of chromosomal instability in Bloom's syndrome. Oncogene.

[44] Davies S.L., North P.S., Dart A., Lakin N.D., Hickson I.D. (2004). Phosphorylation of the Bloom's syndrome helicase and its role in recovery from S-phase arrest. Mol. Cell. Biol..

[45] Do T., Ho F., Heidecker B., Witte K., Chang L., Lerner L. (2008). A rapid method for determining dynamic binding capacity of resins for the purification of proteins. Protein Expr. Purif..

[46] Svergun D.I. (1992). Determination of the regularization parameter in indirect-transform methods using perceptual criteria. J. Appl. Crystallogr..

[47] Svergun D., Barberato C., Koch M.H.J. (1995). CRYSOL—a program to evaluate x-ray solution scattering of biological macromolecules from atomic coordinates. J. Appl. Crystallogr..

[48] Konarev P.V., Volkov V.V., Sokolova A.V., Koch M.H.J., Svergun D.I. (2003). PRIMUS: a Windows PC-based system for small-angle scattering data analysis. J. Appl. Crystallogr..

[49] Rambo R.P., Tainer J.A. (2013). Accurate assessment of mass, models and resolution by small-angle scattering. Nature.

[50] Petoukhov M.V., Svergun D.I. (2005). Global rigid body modeling of macromolecular complexes against small-angle scattering data. Biophys. J..

[51] Pardon E., Laeremans T., Triest S., Rasmussen S.G., Wohlkonig A., Ruf A., Muyldermans S., Hol W.G., Kobilka B.K., Steyaert J. (2014). A general protocol for the generation of Nanobodies for structural biology. Nat. Protoc..

[52] Beresten S.F., Stan R., van Brabant A.J., Ye T., Naureckiene S., Ellis N.A. (1999). Purification of overexpressed hexahistidine-tagged BLM N431 as oligomeric complexes. Protein Expr. Purif..

[53] Pike A., Gomathinayagam S., Swuec P., Berti M., Zhang Y., Schnecke C., Marino F., von Delft F., Renault L., Costa A. (2015). Human RECQ1 helicase-driven DNA unwinding, annealing, and branch migration: Insights from DNA complex structures. Proc. Natl. Acad. Sci. U.S.A..

[54] Bernstein D.A., Zittel M.C., Keck J.L. (2003). High-resolution structure of the E. coli RecQ helicase catalytic core. EMBO J..

[55] Bernstein D.A., Keck J.L. (2003). Domain mapping of Escherichia coli RecQ defines the roles of conserved N- and C-terminal regions in the RecQ family. Nucleic Acids Res..

[56] Ren H., Dou S.X., Rigolet P., Yang Y., Wang P.Y., Amor-Gueret M., Xi X.G. (2007). The arginine finger of the Bloom syndrome protein: its structural organization and its role in energy coupling. Nucleic Acids Res..

[57] Nadanaciva S., Weber J., Wilke-Mounts S., Senior A.E. (1999). Importance of F1-ATPase residue alpha-Arg-376 for catalytic transition state stabilization. Biochemistry.

[58] Kim S.Y., Hakoshima T., Kitano K. (2013). Structure of the RecQ C-terminal domain of human Bloom syndrome protein. Sci. Rep..

[59] Thangavel S., Mendoza-Maldonado R., Tissino E., Sidorova J.M., Yin J., Wang W., Monnat R.J. Jr, Falaschi A., Vindigni A. (2010). Human RECQ1 and RECQ4 helicases play distinct roles in DNA replication initiation. Mol. Cell. Biol..

[60] Velankar S.S., Soultanas P., Dillingham M.S., Subramanya H.S., Wigley D.B. (1999). Crystal structures of complexes of PcrA DNA helicase with a DNA substrate indicate an inchworm mechanism. Cell.

[61] Garcia P.L., Bradley G., Hayes C.J., Krintel S., Soultanas P., Janscak P. (2004). RPA alleviates the inhibitory effect of vinylphosphonate internucleotide linkages on DNA unwinding by BLM and WRN helicases. Nucleic Acids Res..

[62] Buttner K., Nehring S., Hopfner K.P. (2007). Structural basis for DNA duplex separation by a superfamily-2 helicase. Nat. Struct. Mol. Biol..

[63] Marintcheva B., Weller S.K. (2003). Helicase motif Ia is involved in single-strand DNA-binding and helicase activities of the herpes simplex virus type 1 origin-binding protein, UL9. J. Virol..

[64] Zittel M.C., Keck J.L. (2005). Coupling DNA-binding and ATP hydrolysis in Escherichia coli RecQ: role of a highly conserved aromatic-rich sequence. Nucleic Acids Res..

[65] Hura G.L., Budworth H., Dyer K.N., Rambo R.P., Hammel M., McMurray C.T., Tainer J.A. (2013). Comprehensive macromolecular conformations mapped by quantitative SAXS analyses. Nat. Methods.

[66] Rambo R.P., Tainer J.A. (2011). Characterizing flexible and intrinsically unstructured biological macromolecules by SAS using the Porod-Debye law. Biopolymers.

[67] Swan M.K., Legris V., Tanner A., Reaper P.M., Vial S., Bordas R., Pollard J.R., Charlton P.A., Golec J.M., Bertrand J.A. (2014). Structure of human Bloom's syndrome helicase in complex with ADP and duplex DNA. Acta Crystallogr. D Biol. Crystallogr..

[68] Aggarwal M., Sommers J.A., Shoemaker R.H., Brosh R.M. Jr (2011). Inhibition of helicase activity by a small molecule impairs Werner syndrome helicase (WRN) function in the cellular response to DNA damage or replication stress. Proc. Natl. Acad. Sci. U.S.A..

[69] Gyimesi M., Sarlos K., Kovacs M. (2010). Processive translocation mechanism of the human Bloom's syndrome helicase along single-stranded DNA. Nucleic Acids Res..

[70] Manthei K.A., Hill M.C., Burke J.E., Butecher S.E., Keck J.L. (2015). Structural mechanisms of DNA binding and unwinding in bacterial RecQ helicases. Proc. Natl. Acad. Sci. U.S.A..

