# Supplementary material for: Crystal structure of the Bloom's syndrome helicase indicates a role for the HRDC domain in conformational changes
Source: Nucleic Acids Res. 2015 Apr 21;43(10):5221–35. doi: 10.1093/nar/gkv373 (PMC4446433; doi:10.1093/nar/gkv373)
Supplement: SUPPLEMENTARY DATA [file supp_43_10_5221__index.html]

Crystal structure of the Bloom's syndrome helicase indicates a role for the HRDC domain in conformational changes — SUPPLEMENTARY DATA 

# Crystal structure of the Bloom's syndrome helicase indicates a role for the HRDC domain in conformational changes

## SUPPLEMENTARY DATA

**Files in this Data Supplement:**

- SUPPLEMENTARY DATA
- SUPPLEMENTARY DATA
