# Supplementary material for: Crystal structure of the Bloom's syndrome helicase indicates a role for the HRDC domain in conformational changes
Source: Nucleic Acids Res. 2015 Apr 21;43(10):5221–35. doi: 10.1093/nar/gkv373 (PMC4446433; doi:10.1093/nar/gkv373)
Supplement: SUPPLEMENTARY DATA [file supp_gkv373_nar-00084-m-2015-File010.pdf]

Supplementary data.

Table S1 Data collection and refinement statistics

Data collection statistics			
	**Nanobody Complex**	**DNA-complex I**	**DNA-complex II**
Space group	P1	$P4_2$	$P4_2$
Cell dimensions, *a,b,c* (Å)	59.7, 88.4, 95.1	149.5, 149.5, 64.1	151.4, 151.4, 64.5
Angles α,β,γ (°)	69.4, 89.8, 77.1	90, 90, 90	90, 90, 90
Wavelength (Å)	0.968	0.968	0.968
Resolution (Å)	42.9 – 2.8 (2.95 – 2.8)	48.6 – 3.2 (3.42 – 3.20)	19.6-4.7 (5.26-4.70)
R_{merge}	0.1 (0.85)	0.04 (1.5)	0.06 (0.85)
$R_{p.i.m.}$	0.04 (0.38)	0.28 (1.86)	0.03 (0.46)
I/σI	15.9 (2.2)	11.9 (0.6)	10.7 (1.9)
I/σI hk plane[#]	-	12.16 (0.19)	8.4 (0.8)
I/σI l axis[#]	-	37.8 (3.74)	14.9 (5.73)
Completeness (%)	99.0 (98.5)	99.5 (99.7)	98.5 (99.7)
Multiplicity	7.5 (6.1)	3.4 (3.4)	4.4 (4.5)
No. Unique reflections	43258 (6292)	23593 (4216)	7733 (2198)
**Refinement statistics**			
Resolution	42.9 – 2.8 (2.82 – 2.79)	48.6 – 3.2 (3.26 - 3.20)	19.6-4.7 (5.06 – 4.70)
R_{work}/R_{free} (%)	20.6/24.6	22.9/25.9	23.6/29.7
No. atoms			
Protein	11655	5040	5040
DNA	-	582	592
Ligand/ion	56	28	28
Average B factors ($Å^2$)			
All atoms	69.6	179	-
Protein	69.7	178	-
Ligand/ion	50.4	161	-
DNA	-	189	-
Wilson B	67	146	-
R.M.S. deviations			
Bond lengths (Å)	0.002	0.006	-
Bond angles (°)	0.66	1.13	-
Ramachandran plot			
Favoured (%)	97.5	96	-
Allowed (%)	100	100	-
PDBid	4CDG	4CGZ	-

I/σI statistics are for data within 20° of the hk plane and l axis respectively.

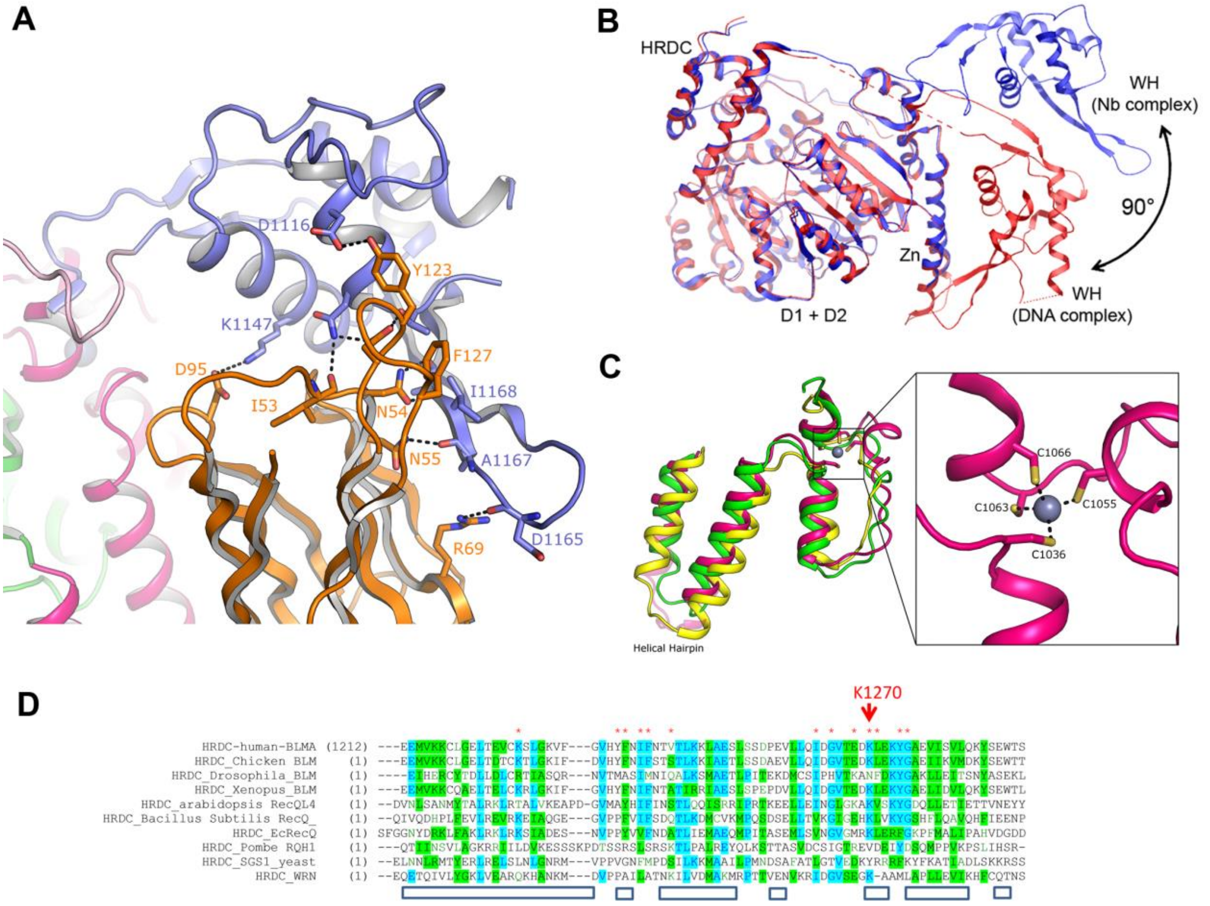

Figure S1 Details of the BLM-nanobody complex. **(A)** The nanobody (orange) binds in a tight cleft between the WH (blue) and Zn^{2+} binding (red) domains and forms multiple polar contacts to residues in the WH domain. **(B)** Superposition of BLM structure in the nanobody complex (PDB:4CDG, blue) and the DNA complex (PDB:4CDZ, red). The nanobody and DNA are not shown. The two structures are virtually superimposable in all regions except the WH domain. **(C)** The Zn-binding domains. The details of the Zn^{2+} binding subdomain differs significantly between BLM (pink), human RECQ1 (green) and *E.coli* RecQ (yellow). Residues at the far end of the helical hairpin are disordered in the nanobody complex but become ordered in the DNA complex, and are shown as a semi-transparent. **(D)** Sequence alignment of HRDC domains of BLM homologues and other RecQ-family proteins. Amino acid residues contacting the RecA domain of BLM are marked with asterisks, and the α-helices are demoted by the rectangles at the bottom.

A

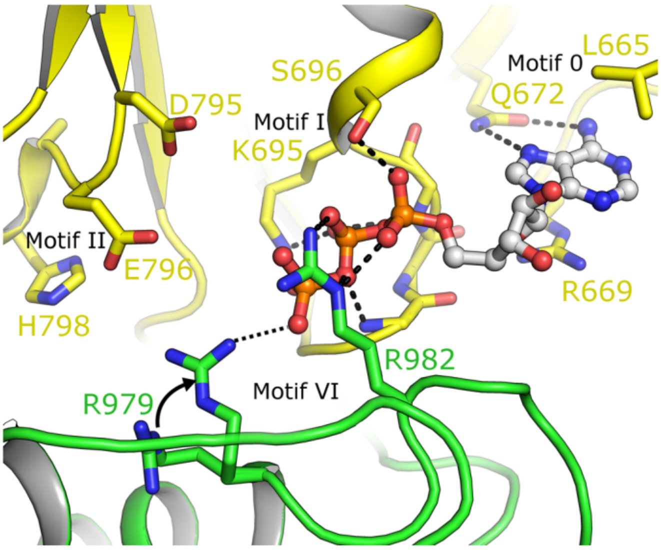

B

```
                                                        Motif 0                 Motif I
601                                                     SRNLK HERFQSLSFP
651   HTKEMMKIFH KKFGLHNFRT NQLEAINAAL LGEDCFILMP TGGGKSLCYQ
                  Motif Ia
701   LPACVSPGVT VVISPLRSLI VDQVQKLTSL DIPATYLTGD KTDSEATNIY
                  Motif Ib                                Motif II
751   LQLSKKDPII KLLYVTPEKI CASNRLISTL ENLYERKLLA RFVIDEAHCV
      Arom.loop                    Mot. III
801   SQWGHDFRQD YKRMNMLRQK FPSVPVMALT ATANPRVQKD ILTQLKILRP
                                                          Mot. IV
851   QVFSMSFNRH NLKYYVLPKK PKKVAFDCLE WIRKHHPYDS GIIYCLSRRE
                     Mot.IVa                               Motif V
901   CDTMADTLQR DGLAALAYHA GLSDSARDEV QQKWINQDGC QVICATIAFG
      (V)                          Motif VI                    (Zn)
951   MGIDKPDVRF VIHASLPKSV EGYYQESGRA GRDGEISHCL LFYTYHDVTR
                     Zn-domain
1001  LKRLIMMEKD GNHHTRETHF NNLYSMVHYC ENITECRRIQ LLAYFGENGF
         (Zn)                          WH domain
1051  NPDFCKKHPD VSCDNCCKTK DYKTRDVTDD VKSIVRFVQE HSSSQGMRNI
                  (WH domain)
1101  KHVGPSGRFT MNMLVDIFLG SKSAKIQSGI FGKGSAYSRH NAERLFKKLI
                   (WH domain)
1151  LDKILDEDLY INANDQAIAY VMLGNKAQTV LNGNLKVDFM ETENSSSVKK
                        HRDC domain
1201  QKALVAKVSQ REEMVKKCLG ELTEVCKSLG KVFGVHYFNI FNTVTLKKLA
                        (HRDC)
1251  ESLSSDPEVL LQIDGVTEDK LEKYGAEVIS VLQKYSEWTS PAEDSSPG
```

Figure S2 Modelling of ATP binding by conserved helicase motifs. **(A)** Modelling of ATP into the nucleotide binding site reveals possible interactions between the gamma phosphate and the conserved arginine R979 from helicase motif VI.
(B) Conserved Superfamily-2 helicase sequence motifs in BLM. The Zn, WH and HRDC domains are also delineated. Residues mentioned in the text are marked in red. For sequence alignment with other RecQ proteins, see Fig. S7 and Vindigni et al (2010), Biophysical Chemistry *149*, 67-77.

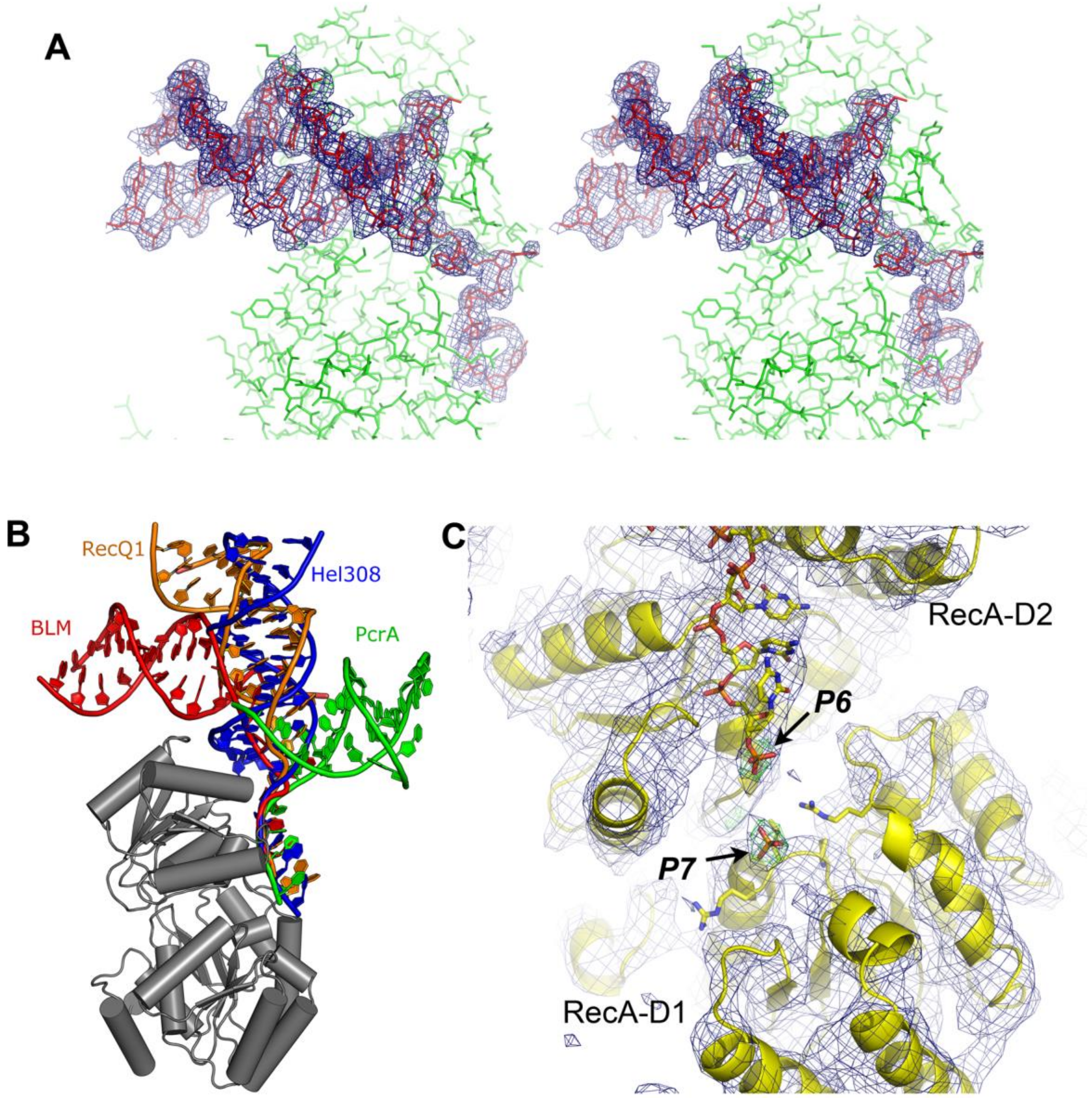

Figure S3 BLM-DNA complex structures. **(A)** Complex I. Stereo view of the electron $2F_o$-$1F_c$ electron density map contoured at 1.4 σ around the DNA molecule. The DNA includes a 12-bp duplex with a 3' ssDNA overhang of 5 nucleotides. **(B)** Comparison of the DNA orientation in four helicase: DNA complexes. The double stranded region of the DNA can be seen to vary significantly even between close homologues, whilst the single stranded overhang can be seen in all complexes to converge around the DNA binding motifs of the second RecA like domain.**(C)** Electron density map of the 4.8 Å BLM DNA complex II crystals contoured at 1.3σ (blue $2F_o$-$1F_c$ type map), and 4.0σ (green F_o-F_c type map). The position of two additional phosphates (which were excluded from the model during map calculations) can be clearly seen close to the first RecA domain.

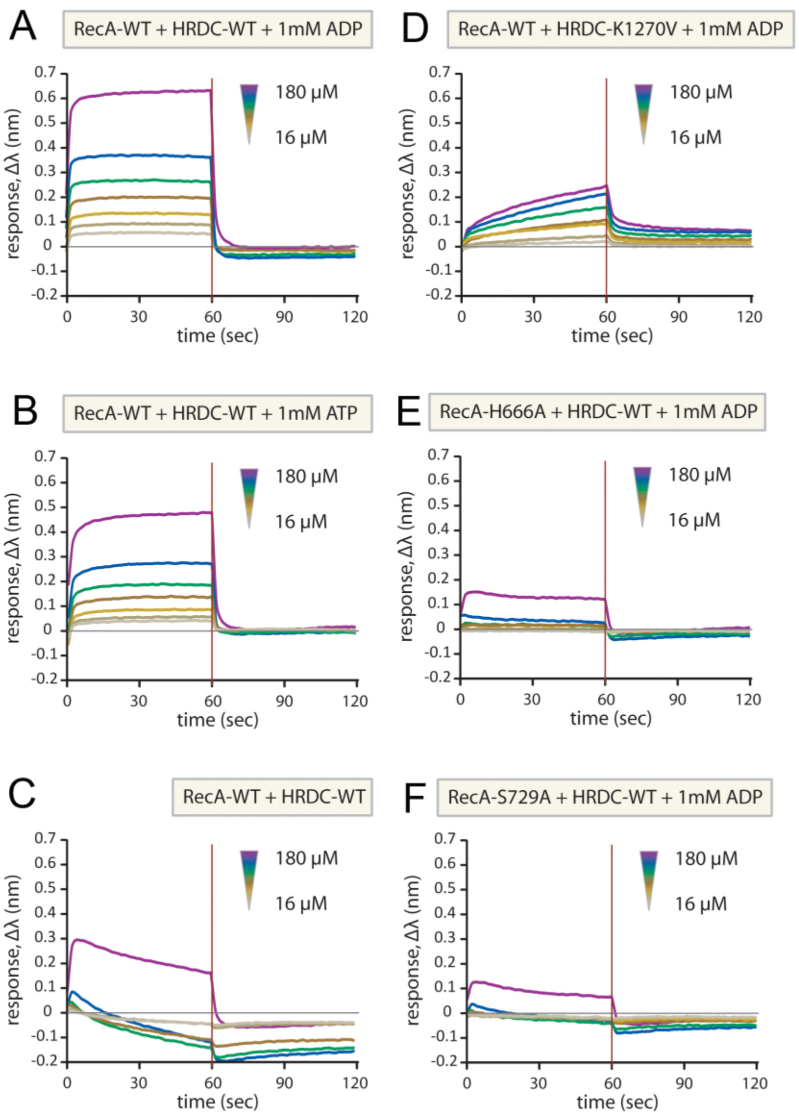

Figure S4 Binding of isolated WT and mutant HRDC and RecA domains measured by biolayer interferometry (BLI). Biotinylated HRDC domains (WT or K1270V mutants as indicated) were immobilized to the sensor tips, which were then dipped in solutions containing the RecA domain proteins in concentrations ranging from 0 to 180 μM. Panels A and C are the same as Fig 4 of the main text.

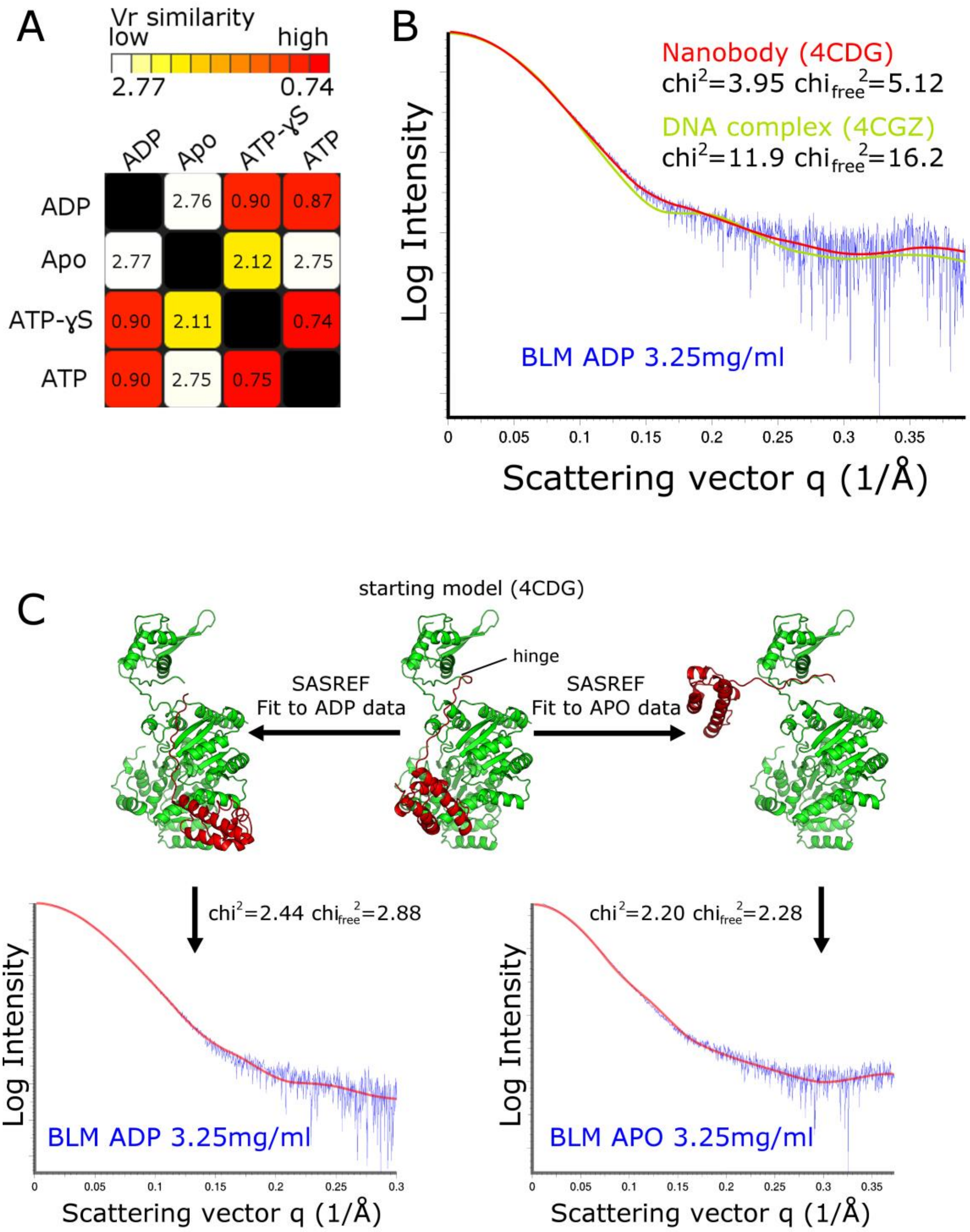

Figure S5 Analysis of the BLM conformational change by SAXS. **(A)** Quantitative comparison matrix of the different BLM SAXS datasets evaluated by the program Vr. **(B)** Comparison of the calculated scattering curves for the BLM nanobody complex (red line with contribution of the nanobody omitted) and the BLM DNA complex (green line with contribution of the BNA omitted), against the experimental data for the ADP bound form. The chi^2 chi_{free}^2 values for the nanobody complex are slightly better than those for the DNA complex, suggesting that in the absence of DNA the WH domain is closer to the conformation seen in the nanobody complex. **(C)** Rigid body modelling of the conformation of the HRDC domain using the program SASREF. The nanobody structure (upper centre panel) was used as a starting model which was refined against the SAXS data as a two body system with a single constraint to keep residues on either side of the hinge region indicated within 6 Å. The

final confirmation of the domains is shown in the upper left (ADP data) and right (APO data) panels, with the fit to the raw data and chi^2 chi$_{free}^2$ values shown in the lower panel.

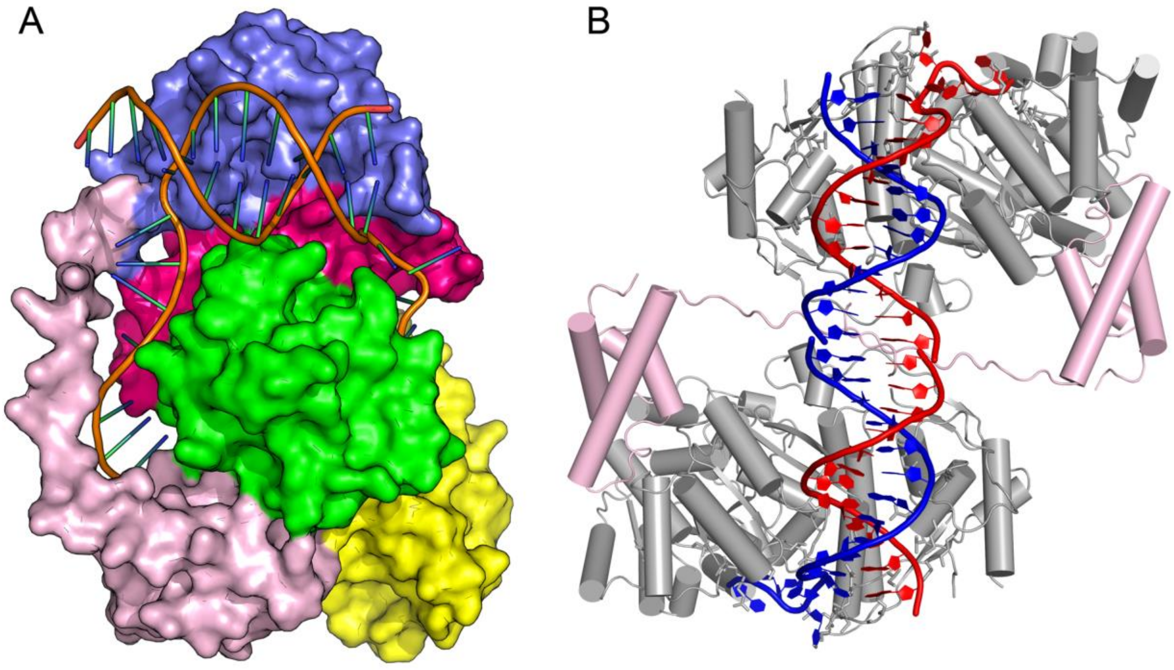

Figure S6 Possible alternative roles for the HRDC domain when bound to double Holliday junctions. **(A)** Surface representation of a model of BLM in complex with DNA containing extended 3’ and 5’ overhangs. The loop connecting the HRDC and WH domains is assumed to adopt a similar conformation to that observed in the nanobody complex and forms a hole in the protein surface through which it is possible to thread single stranded but not double stranded DNA. **(B)** The BLM DNA complex viewed along a crystallographic two-fold symmetry axis. The arrangement of the symmetry related molecules resembles what might be expected when two BLM molecules bound to opposite strands of a double Holliday junction are branch migrated together. We note that in this scenario the HRDC domain and the extended linker form the primary region in which the two molecules contact each other.

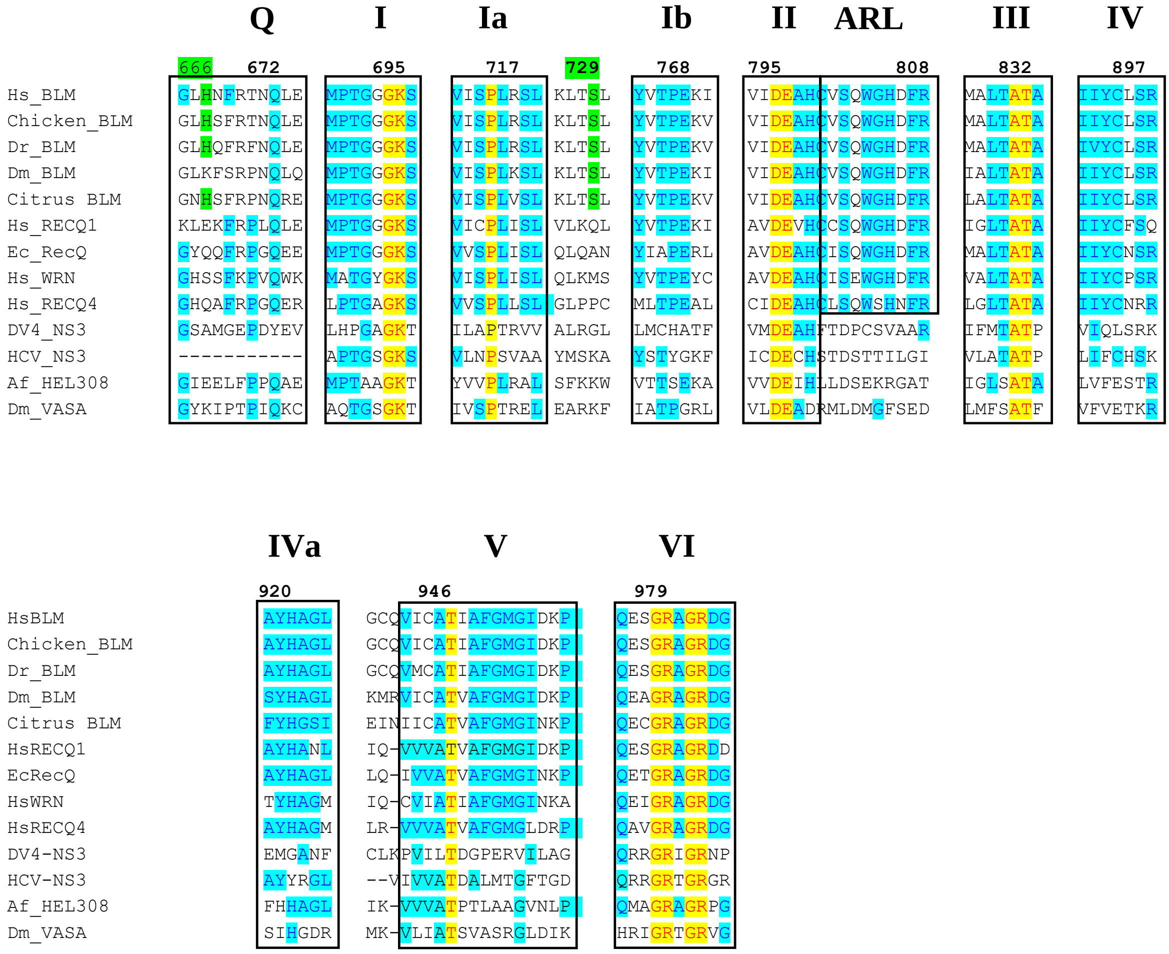

Fig S7 Alignment of helicase signature motifs in LM and other SF2 helicases.

The proteins, listed from the top, are: BLM orthologues from humans (Hs), Chicken, Zebrafish (Dr), Fruit fly (Dm) and Citrus clementina; other RecQ family proteins: human RECQ1, *E. coli* RecQ, Human WRN, Human RECQ4; other Superfamily 2 helicases: Dengue virus NS3, Hepatitis C virus NS3, Archaeoglobus fulgidus HEL308, and Drosophila VASA.

The numbers above the sequences indicate the positions of selected residues in HsBLM. Roman numerals designate the conserved helicase motifs. ARL is the aromatic loop. Residues H666 and S729 (highlighted in green), involved in contacts with the HRDC domain, are conserved among BLM orthologues but less so in other helicases.
